# Oral Microbiota in Infants Fed a Formula Supplemented with Bovine Milk Fat Globule Membranes - A Randomized Controlled Trial

**DOI:** 10.1371/journal.pone.0169831

**Published:** 2017-01-18

**Authors:** Niklas Timby, Magnus Domellöf, Pernilla Lif Holgerson, Christina E. West, Bo Lönnerdal, Olle Hernell, Ingegerd Johansson

**Affiliations:** 1 Department of Clinical Sciences/Unit of Pediatrics, Umeå University, Umeå, Sweden; 2 Department of Odontology/Unit of Pedodontics, Umeå University, Umeå, Sweden; 3 Department of Nutrition, University of California, Davis, United States of America; 4 Department of Odontology/Unit of Cariology, Umeå University, Umeå, Sweden; Istanbul Universitesi Dis Hekimligi Fakultesi, TURKEY

## Abstract

**Background:**

In a recent study, supplementation of infant formula with milk fat globule membranes (MFGM) decreased the incidence of otitis media in infants <6 months of age.

**Objectives:**

The aim of the present study was to characterize the oral microbiota in infants fed MFGM-supplemented formula and compare it to that of infants fed standard formula or breast milk.

**Methods:**

In a prospective double-blinded randomized controlled trial, exclusively formula-fed infants <2 months of age were randomized to be fed experimental formula (EF, n = 80) with reduced energy and protein and supplemented with a bovine MFGM concentrate, or standard formula (SF, n = 80) until 6 months of age. A breast-fed reference (BFR, n = 80) group was also recruited. The oral microbiota was analyzed at 4 (n = 124) and 12 (n = 166) months of age using Illumina MiSeq multiplex sequencing and taxonomic resolution against the HOMD 16S rDNA database of oral bacteria.

**Results:**

Species richness in the oral samples did not differ between the EF and SF groups, but partial least square modeling identified a few taxa that were significantly associated with being in either group, e.g. lower level of *Moraxella catarrhalis* in the EF group. Infants in the BFR group had significantly lower species richness at 4 months of age and their microbiota pattern differed markedly from the formula-fed groups.

**Conclusions:**

Supplementation of infant formula with MFGM yielded moderate effects on the oral microbiome. *Moraxella catarrhalis* was less prevalent in infants fed EF than in those fed SF and may be associated with the decrease in otitis media seen in the same group.

## Introduction

Breast-feeding, as compared to formula-feeding, reduces the risk of infections including acute otitis media (AOM), even in high-income countries [1–3]. This is probably due to antimicrobial factors in human milk, such as immunoglobulins, antibacterial proteins/peptides, leukocytes and probiotic bacteria [4], but also ligand epitopes for attachment/clearance of opportunistic and commensal bacteria, such as oligosaccharides (free or on glycoproteins or lipids), lactose, and proteins/peptides [5]. Thus, protection against infections from milk could be mediated by direct effects on pathogens, or by factors influencing the ecology of the gastrointestinal (GI) tract. The latter is supported by the fact that the complex microbiomes of the GI tract (mouth and gut) differ distinctly between breast-fed and formula-fed infants [6, 7].

In the development of infant formulas with the aim to narrow the gap between breast-fed and formula-fed infants, the milk fat globule membrane (MFGM), a small but in terms of biological activities significant milk fraction, has gained interest since it seems to have several health promoting effects including defense against infections [8–12]. This membrane fraction includes several components with antimicrobial effects including gangliosides [13], oligosaccharides [14] and the glycoproteins butyrophilin, lactadherin and mucins [9, 14]. The MFGM fraction thus has potential to affect bacteria attachment, colonization, clearance and metabolism in the oral cavity and the gut [15]. Historically, the MFGM has been discarded with the milk fat in the manufacturing of infant formula as blends of vegetable oils have been used as the fat source.

We have, in a recently performed randomized controlled trial, evaluated health effects in infants fed an experimental formula (EF) supplemented with a bovine MFGM fraction, compared to infants fed standard formula (SF) [16]. Infants fed the EF had a significantly lower incidence of infections leading to hospital stay or prescription of antibiotics during the first 6 months, and the most common diagnosis was acute otitis media (AOM). The EF group also had lower use of antipyretics and differed in antibody titers against pneumococcal vaccine [17]. We concluded that MFGM supplementation provided a protective effect against infections, especially AOM. Bacterial colonization of the upper airways has been suggested to be important for the pathogenesis in AOM [18], and not only nasopharynx cultures [19–21] but also cultures from saliva [22, 23] at onset of AOM correlate to otopathogens

The aim of this study, which was part of the above described clinical trial [16], was to characterize and compare the oral microbiome in infants fed MFGM supplemented formula with that of infants fed standard formula or a breast-fed reference group. Our hypothesis was that MFGM exposure would influence the composition of the oral microbiota and that the altered microbiota would contribute to a reduced risk of AOM.

## Materials and Methods

### Ethics Statements

The study was approved by the Regional Ethical Review Board in Umeå, Sweden (Dnr 07-083M), and conducted according to the principles in the Declaration of Helsinki. Written informed consent was obtained from all caregivers. The trial was registered at clinicaltrials.gov as NCT00624689.

### Study Subjects

**Fig 1:**
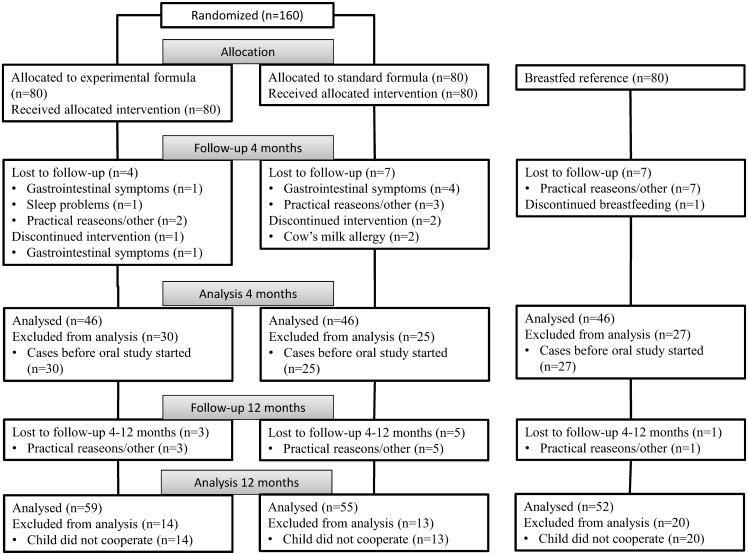
CONSORT 2010 Flow Diagram.

Exclusively formula-fed infants (n = 160) were recruited consecutively from March 2008 to February 2012 and randomized to receive standard (SF, BabySemp 1^®^, Semper AB, Sundbyberg, Sweden) or experimental (EF) formula until 6 months of age as described previously [16]. The EF had a reduced protein concentration (1.20 vs. 1.27 g/100mL), a reduced energy density (60 vs. 66 kcal/100 mL) and was supplemented with a bovine MFGM concentrate (Lacprodan MFGM-10^®^, Arla Foods Ingredients, Viby, Denmark). A breast-fed reference group (BFR) consisting of 80 infants was also recruited. The inclusion criteria were <2 months of age, gestational age at birth 37–42 weeks, birth weight 2 500–4 500 g, absence of chronic illness, and exclusively formula-fed at baseline, and for the breast-fed reference group that the infant was exclusively breast-fed at inclusion and that the mother had the intention to breast-feed until 6 months. All caregivers were recommended to not give any taste portions of complementary foods until the infant was 4 months of age, and then only small amounts up to 6 months of age. Weight and length were assessed at visits at baseline, 4, 6 and 12-months of age. Compliance to the diet regimen and information on intake of taste portions, probiotic drops and antibiotics, as well as disease symptoms, medical contacts and given medications were assessed by a prospective parental diary and from medical records. As described in detail previously [16], the number of included infants was based on a pre-study power analysis for the original study to detect a difference of 0.5 SD in the main outcomes weight-for-age and cognitive score at a significance level of 0.05. Data collection and analyses followed the original project plan shown as supplemental information. The CONSORT 2010 flow diagram of the study is shown in Fig 1.

### Oral Microbiota Sampling

At 4 and 12 months of age, the buccal mucosa, tongue, and alveolar ridges were swabbed carefully using sterile cotton swabs (Applimed SA, Chatel-St-Denis, Switzerland). At 12 months of age, swabbing also included the teeth. The swab samples for the child were immediately pooled into Eppendorf tubes (Sarstedt, Nümbrecht, Germany) with 200 μl TE-buffer (10 mM Tris, 1 mM EDTA, pH 7.6). In addition, at both 4 and 12 months of age, approximately 1 mL saliva was collected from the child, into ice-chilled, sterile test tubes and lactobacilli were cultivated as described previously [24]. All samples were obtained between 1–3 h (mean 2 h) after the latest meal, and stored at −80°C.

### DNA Extraction

Genomic DNA was extracted from the swab samples using the GenElute^™^ Bacterial Genomic DNA kit (Sigma-Aldrich, St. Louis, MO, USA) according to the manufacturer´s recommendations. Briefly, the samples were (*i*) centrifuged for 5 min at 13 000 rpm, (*ii*) lysed in a lysis buffer with addition of lysozyme and mutanolysin for 30 min at 37°C, (*iii*) treated with RNase for 2 min at room temperature followed by Proteinase K for 10 min at 55°C, (*iv*) mixed with ethanol and transferred to the binding column, and (*v*) washed and eluted in 100 μl elution buffer. All these ingredients were included in the DNA extraction kit. The quality and quantity of the DNA were evaluated using a Nanodrop 1000 spectrophotometer (Thermo Scientific, Wilmington, DE) to meet the standard set by the sequencing facility, namely, an OD 260/280 ratio ≥1.8.

### 16S rDNA Sequencing and Data Processing

PCR amplification of the V3-V4 hypervariable region of the bacteria 16S rRNA gene using the forward primer, 341F (CCTACGGGAGGCAGCAG) and the reverse primer, 806R (GGACTACHVGGGTWTCTAAT), and sample library preparation, and Illumina MiSeq sequencing was conducted at the Forsyth Sequencing Facility (HOMINGS, http://homings.forsyth.org/index2.html). Sequences have been uploaded at www.figshare.com doi: 10.6084/m9.figshare.3422962

Pair-ended reads were merged using FLASH (http://ccb.jhu.edu/software/FLASH), and merged reads and barcodes were matched using the python script fastqCombinePairEnd.py. Quality filtering of retained sequences was done using Quantitative Insights into Microbial Ecology (QIIME, version 1.8.0). Sequences with a minimum length of 300 base pairs after primer sequence removal, with correct barcode sequences and primer sequences, and not meeting default quality filtering criteria for homopolymers and quality scores in QIIME were retained. Chimeric sequences, as identified by UCLUST, were removed. Retained sequences were clustered into operational taxonomic units (OTUs) at 97% similarity to the Human Oral Microbiome Database (HOMD) (http://www.homd.org/) and taxonomically named by BLAST to the same database for one representative sequence per OTU. The HOMD is a curated database holding 700 named species and taxa identified from 16S rRNA gene sequence analysis of oral isolates and cloning studies. In HOMD, approximately 54% are named species, 14% unnamed (but cultivated) and 32% are known only as uncultivated phylotypes. Each 16S rRNA phylotype is given a unique Human Oral Taxon (HOT) number [25].

### Data Analyses

IBM SPSS Statistics (version 22.0; IBM Corporation, Armonk, NY, USA) was used for descriptive analyses and univariate testing of differences and associations. Normally (confirmed by Shapiro-Wilk´s tests) distributed variables were presented as means with 95% confidence intervals and differences between means tested with ANOVA. For non-normally distributed variables medians with range were calculated, and the Kruskal-Wallis test used to test differences between groups. HOT taxa prevalence were highly skewed with >50% of the subjects lacking detection. Therefore, detection frequency (% children) and mean prevalence (% of total number of reads) in the three feeding groups are presented, together with median prevalence among those with detected taxa. Differences in distributions between groups were tested using Chi-square test. For comparisons between HOT taxa a p-value ≤0.008 (accounting for multiple testing by the False discovery rate), and for other variables a p-value <0.05 were considered statistically significant.

Rarefaction curves were calculated to compare microbial richness among the three feeding group samples, and principal coordinate analysis (PCoA) to compare the phylogenetic diversity (β diversity) and search for clustering of samples based on the OTU assignment by QIIME. In addition, multivariate principal component analysis (PCA) and partial least square (PLS, SIMCA P+, version 12.0, Umetrics AB, Umeå, Sweden) regression with assigned taxa and potential confounders (mode of delivery, sex, anthropometric measures and lactobacilli by culture) in the independent block were done. These analyses searched for clustering of samples based on taxonomic assignment with addition of potential confounders (PCA) and to identify taxa associated with the EF, SF or BFR groups (PLS regression) as previously described [24]. For PCA and PLS regression, variables were auto-scaled to unit variance, and cross-validated predictions of Y (here feeding groups) were calculated. Clustering of subjects is displayed in a score-loading plot, and the importance of each x-variable is displayed in a loading plot. Variables with a 95% confidence interval for the PLS correlation coefficient that did not include zero, were considered statistically significant. Besides the explanatory R^2^-value, PLS regression provides a cross validated predictive value (Q^2^) of the model.

## Results

Of the infants included in the basic trial, sequencing of the oral microbiota was performed in 64%, 56% and 49% of the 4 months old, and 81%, 81% and 72% of the 12 months old infants in the EF, SF and BFR groups, respectively. The reason for the lower proportions among 4 month olds was that oral sampling was introduced when the basic trial was already going on, and in 12 months old infants it was due to the child not cooperating. Similar to the previous report [16], mean length growth velocity from birth to 1 year of age was lower (p<0.002) in the BFR group compared to the formula-fed groups in the subsample in the present study with no difference between the EF and SF groups (Table 1). Other characteristics, including sex, weight gain, use of antibiotics and probiotics, did not differ significantly among the groups at either age, whereas the proportion born by Caesarean section tended to be higher among formula-fed than breast-fed infants (p = 0.087, Table 1).

**Table 1:** Study group characteristics by age and feeding regimen. Differences in group means were tested with ANOVA and for distributions in groups by chi-square test for the 4- and 12-month olds, respectively.

	4 months old				12 months old			
	EF	SF	BFR	p-value groups	EF	SF	BFR (at 4 month)	p-value groups
Numbers in basic study^a^	76	73	73	−	73	68	72	−
Numbers with sequencing^b^	46	41	37	−	59	55	52	−
Boys^b^ (%)	52	56	40	0.387	49	49	48	0.993
Length^b^ (cm)	64 (64–65)	64 (63–65)	64 (63–65)	0.555	76.5 (75.8–77.1)	76.0 (75.4–76.7)	75.5 (74.9–76.1)	0.108
Length gain 0–12 mo^b^, cm	−	−	−	−	26.2 (25.5–26.9)	25.3 (24.8–25.9)	24.6 (24.0–25.2)	0.002
Weight^b^ (kg)	6.9 (6.7–6.9)	6.9 (6.6–7.2)	6.7 (6.5–6.9)	0.450	10.2 (9.8–10.5)	10.1 (9.8–10.4)	9.9 (9.6–10.1)	0.131
Weight gain 0–12 mo, kg	−	−	−	−	6.7 (6.4–7.0)	6.6 (6.2–6.9)	6.2 (5.9–6.5)	0.062
Caesarean section (%)	18.8	17.5	7.5	0.087	−	−	−	−
Probiotic drops^c^ (%)	6.5	2.4	13.5	0.165	0	0	0	−
Antibiotics^d^ (%)	0	0	0	−	13.3^d^	10.2^d^	6.7^d^	0.627
Total number of quality filtered sequences (mean)	54 459	59 600	58 613	0.322	51 896	48 858	47 108	0.336

^a)^ Remaining infants out of 80 included 0–2 mo old infants at study start. Those who left between recruitment and study start did this for time constraints.

^b)^ The numbers refers to infants with oral swabs analyzed for Illumina MiSeq sequencing of the v3-v4 hypervariable regions of 16S rDNA. The lower numbers at 4 month, compared to numbers included in the basic study, is due to that the basic study was running when inclusion to the present study began. The lower numbers at 12 mo is due to that the child did not cooperate for swabbing.

^c)^ These children (5, 1, 3 infants, respectively) reported having taken probiotic drops (*L*. *reuteri*) during the last several months. No significant difference was seen in phyla, genera or species prevalences between those who reported intake and those who reported no intake.

^d)^ These children (3, 5, 6 infants, respectively) had taken antibiotics once between 7–12 mo of age but with no closer specification when it was taken. No significant difference was seen in phyla, genera or species prevalences between those who reported intake and those who reported no intake.

### Overall Sequencing Results

Sequencing of the oral swab sample from one 12 months old infant failed. For the remaining 290 samples (124 samples from 4months old and 166 from 12 months old infants), 15 274 247 sequences passed the QIIME quality filtering steps. The mean (95% CI) number of filtered sequences per sample were at 4 months of age 57 399 (54 398–60 399) and at 12 months of age 49 164 (46 493–51 835) with no difference between feeding groups at either age (Table 1). The sequence length of filtered sequences varied from 312 to 430 bp (average 416 bp).

The 15 274 247 sequences were clustered into 469 OTUs at 97% similarity against the HOMD database. Of these, 289 were represented with ≥15 sequences per OTU. The 289 OTUs matched into 234 unique HOT taxa of which a few were in a cluster of highly similar species, *i*.*e*. the *Streptococcus oralis* cluster (*S*. *oralis*, *S*. *mitis*, *S*.*mitis bv2*, *S*. *infantis*), the *Streptococcus parasanguinis* II cluster (*S*. *parasanguinis* II, *Streptococcus sp*. HOT065, and *Streptococcus sp*. HOT067), and the *Streptococcus salivarius* cluster (*S*. *salivarius*, *S*. *vestibularis*). Of 234 unique HOT taxa, 202 were present in 4 months old and 229 in 12 months old infants. The 234 unique HOT taxa/clusters represented 10 phyla/divisions (*Actinobacteria*, *Bacteriodetes*, *Firmicutes*, *Fusobacteria*, *GNO2*, *Proteobacteria*, *SR1*, *Synergistes*, *Tenericutes and TM7*), and 64 genera (S1 Table).

At 4 months of age, 98% of the sequences were found in the phyla *Firmicutes* (77%), *Actinobacteria* (11%), *Proteobacteria* (5%), *and Bacteriodetes* (5%). At 12 months of age, the proportions were lower for *Firmicutes* (60%), higher for *Proteobacteria* (18%) but similar for other phyla. Similarly, at 4 months of age, 98% of the sequences were found in 10 genera (*Streptococcus* (64%), *Rothia* (9%), *Veillonella* (8%), *Neisseria* (4%), *Alloprevotella* (3%), *Gemella* (3%), *Prevotella* (2%), *Actinomyces* (2%), *Granulicatella* (2%), *and Haemophilus* (1%). The most striking differences between 4 and 12 months of age were a decrease in *Streptococcus* (to 46%), an increase in *Neisseria* (to 13%), and appearance of *Fusobacteria* (3%), *Leptotrichia* (1%), and *Porphyromonas* (1%). Full lists of detection prevalence (% of the total number of sequences) in 4 and12 months old infants together with p-values are presented for phyla and genera (S2 Table) and for species/phylotypes (S2 Table).

### Core Microbiome at 4 and 12 Months of Age

Twenty-two HOT taxa were identified in ≥95% of all 4 and 12 months old infants. These represented 9 taxa in Streptococcus, 3 in *Streptococcus* clusters, 2 in Veillonella, and 1 each in *Actinomyces*, *Alloprevotella*, *Gemella*, *Granulicatella*, *Haemophilus*, *Neisseria*, *Prevotella*, and *Rothia* (S2 Table).

### Oral Microbiota by Feeding Mode

**Fig 2:**
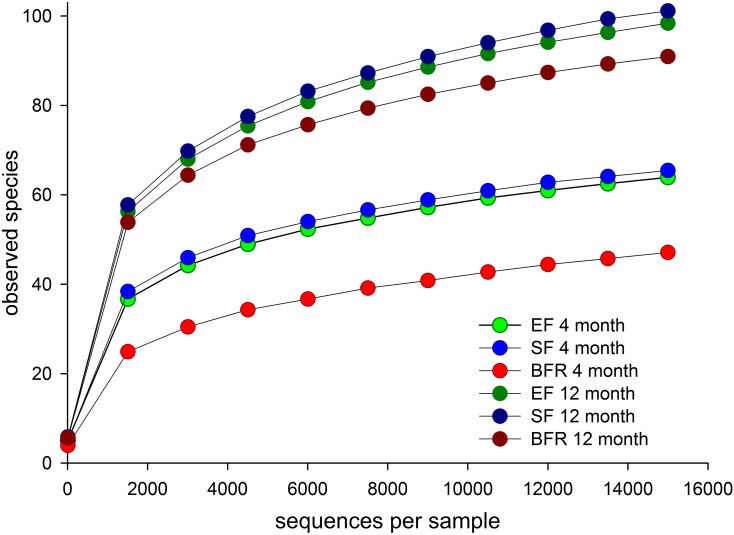
Rarefaction curves showing species richness. Mean numbers of OTUs per subject are shown in the EF, SF, and BFR groups at 4 and 12 months of age, respectively.

Species richness (rarefaction curves) among 4 and 12 months old infants in the EF, SF or BFR groups are shown in Fig 2. The species richness did not differ between the EF and SF group at 4 or 12 months of age. However, at 4 months of age, the BFR group had significantly lower species richness than the formula-fed groups. At 12 months, they still tended to have lower species richness than formula-fed groups but the difference was not statistically significant.

**Fig 3:**
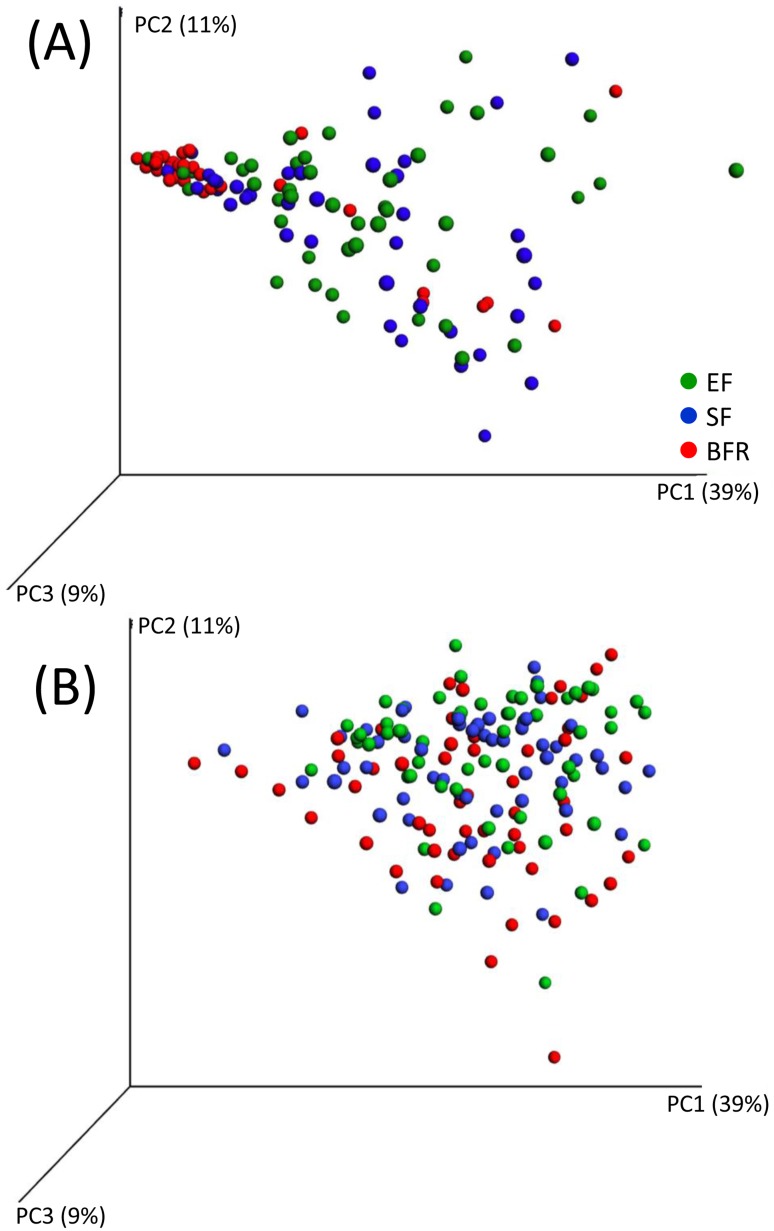
Weighted PCoA plot of QIIME identified OTUs. (A) shows plot for 4 months and (B) for 12 months old infants. Component numbers with % variance explained are indicated at each of the axes.

**Fig 4:**
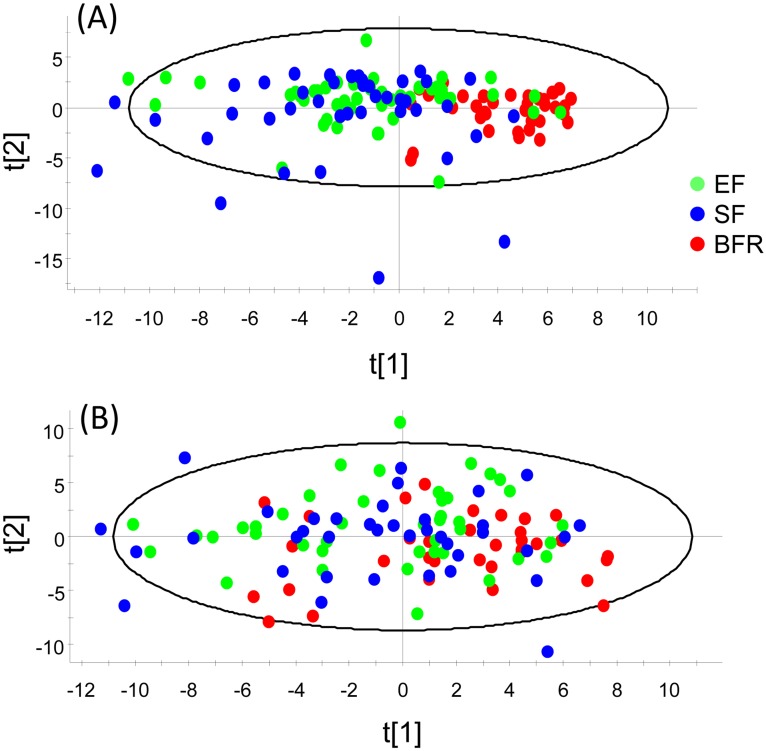
PCA score plot. The figure provides a map of how infants relate to each other by feeding type based on a model including genera and species/phylotypes identified by HOMD blasting after exclusion of OTUs with <15 sequences, sex, mode of delivery, anthropometric measures, and lactobacilli by culture at (A) 4 months and (B) 12 months of age. t1 and t2 are scores for the new PCA created variables summarizing the x variables. Each observation has a t1 and t2 value.

PCoA modeling of the 469 original OTUs at 4 months tended to cluster BFR infants together, whereas EF and SF infants were scattered (Fig 3A). At 12 months of age, clustering had disappeared (Fig 3B). PCA modeling of sex, mode of delivery, anthropometric measures, lactobacilli by culture and the 64 genera, and 232 HOT assigned species/phylotypes (after exclusion of OTUs with <15 sequences) at 4 (Fig 4A) and 12 months of age (Fig 4B), respectively, confirmed the PCoA plot employing unassigned OTUs only, i.e. in Fig 3A and 3B.

**Fig 5:**
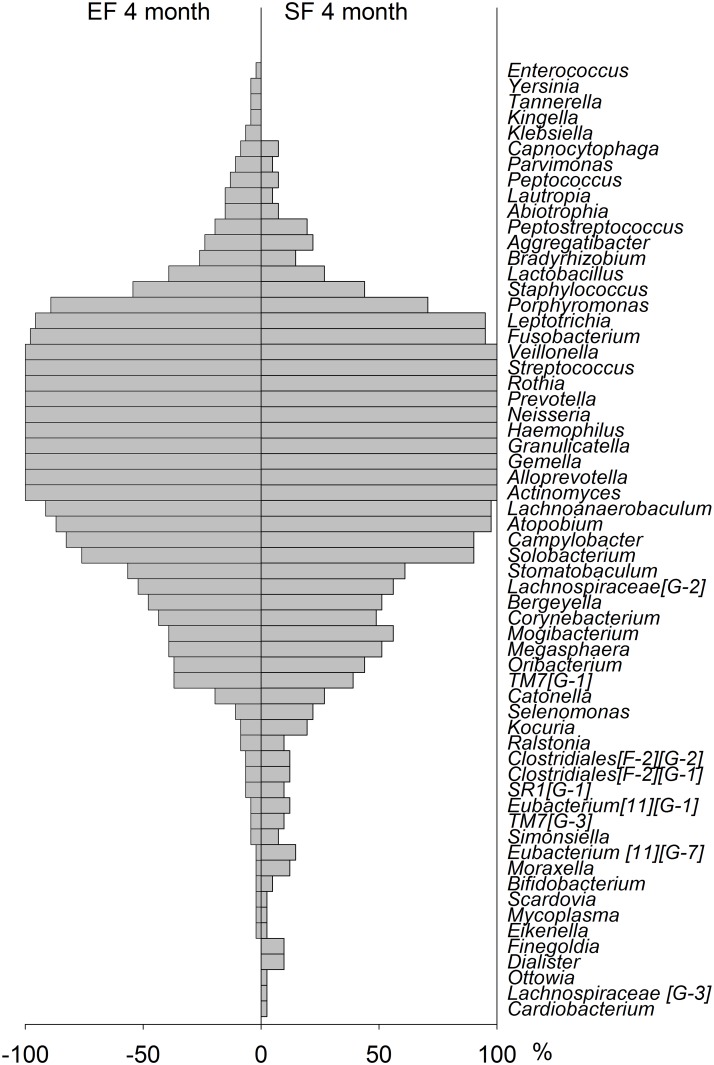
Bilateral bar graph in 4months old infants fed EF versus SF. Detection prevalence is shown as % infants where a species was found.

**Fig 6:**
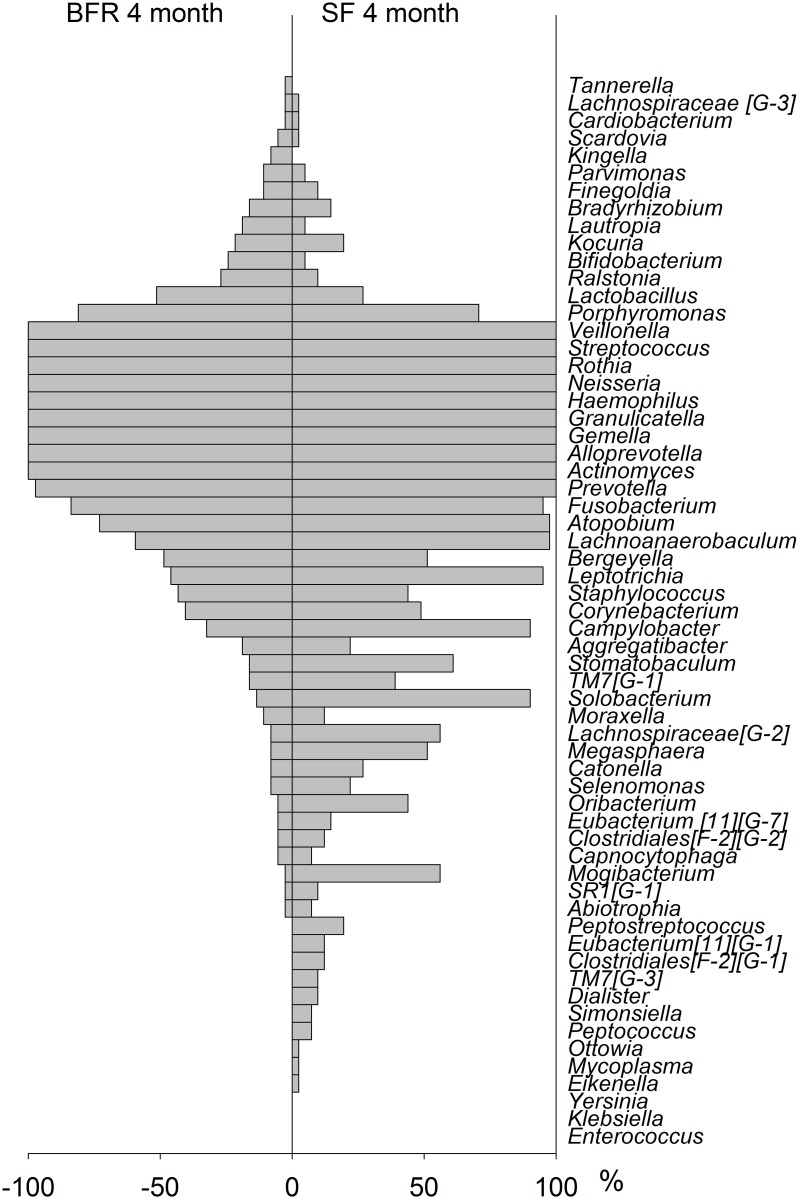
Bilateral bar graph in BFR versus SF fed 4 months old infants. Detection prevalence is shown as % infants where a species was found.

At 4 months of age, the detection prevalence pattern, i.e. % infants where a taxa was found, showed a high degree of symmetry for the EF and SF groups, though with single differences as displayed in the bilateral bar graph in Fig 5. In contrast a markedly different pattern was seen when comparing BFR and SF infants (Fig 6).

**Fig 7:**
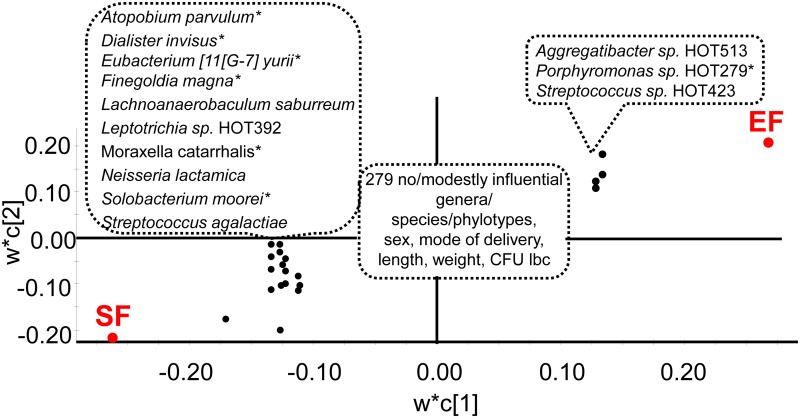
PLS loading scatter plot with being in the EF or SF group as dependent variables. The model included genera and species/phylotypes identified by HOMD blasting after exclusion of OTUs with <15 sequences, sex, mode of delivery, anthropometric measures, and lactobacilli by culture as the independent block. w describes the PLS weights from the combination of the original variables in the X-swarm and c the same for the Y-swarm. Taxa with a statistically significant PLS correlation coefficient ≥0.1 are indicated. * after a taxa denotes that the corresponding genus was influential. For full list and univariate p-values see S1 and S2 Tables.

PLS modeling of the 4 month old infants fed EF or SF (feeding mode as dependent variables) and the same independent block as in the PCA (Fig 4A), identified 2 significant components with a cross validated predictive power (Q^2^) of 15%. Higher levels of 3 HOT-taxa with statistically significant PLS correlation coefficients ≥0.1 (*Aggregatibacter sp*. HOT513, *Porphyromonas sp*. HOT279, *Streptococcus sp*. HOT423) were most strongly associated with being in the EF group (Fig 7) together with 5 taxa with weaker, but statistically significant, correlations for being in the EF group (S3 Table). Being in the SF group was significantly associated with higher levels of 10 HOT-taxa (*Atophium parvulum*, *Dialister invisus*, *Eubacterium yurii*, *Finegolda magna*, *Lachnoanaerobaculum saburreum*, *Leptotrichia sp*. HOT392, *Moraxella catarrhalis*, *Neisseria lactamica*, *Solobacterium moorei*, *and Streptococcus agalactiae*) (Fig 7). At 12 months of age the PLS regression model was considerably weaker (cross-validated predictive power (Q^2^) of 5%) and the HOT-taxa influential for being in the EF versus SF group at 4 months of age did not remain influential (S3 Table).

**Fig 8:**
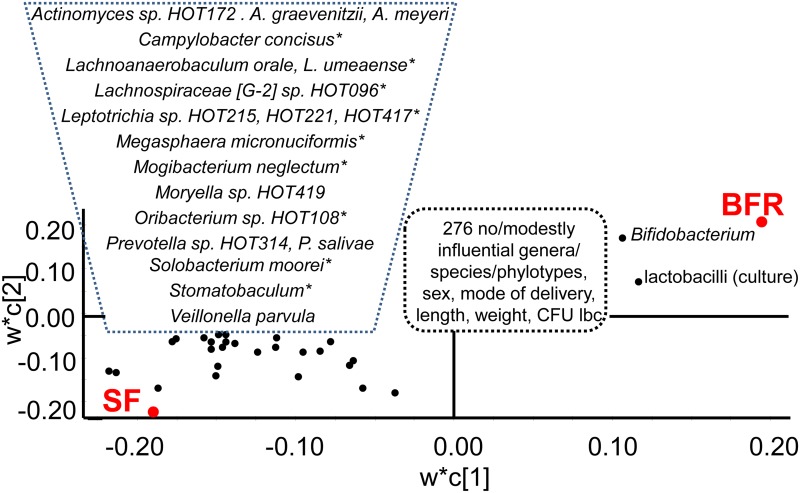
PLS loading scatter plot with being in the BFR or SF group as dependent variables. The model included genera and species/phylotypes identified by HOMD blasting after exclusion of OTUs with <15 sequences, sex, mode of delivery, anthropometric measures, and lactobacilli by culture as the independent block. w describes the PLS weights from the combination of the original variables in the X-swarm and c the same for the Y-swarm. Taxa with a statistically significant PLS correlation coefficient ≥0.1 are indicated. * after a taxa denotes that the corresponding genus was influential. For full list and univariate p-values see S1 and S2 Tables.

The taxa detection pattern differed markedly between infants in the BFR and SF groups at 4 months of age, and PLS regression yielded a strong model (cross validated predictive power (Q^2^) 57%). Being breast-fed was mainly characterized by lactobacilli by culture, *Bifidobacterium* (*B*. *breve*), *Lactobacillus* (*L*. *rhamnosus* and *L*. *gasseri*), *Kingella oralis*, *Ralstonia (Ralstonia sp*. HOT 406), and being a girl (Fig 8, S3 Table). The oral microbiota of 4 months old infants fed SF was characterized by a wide panel of Gram-positive and Gram-negative HOT-taxa (Fig 8, S3 Table). When the infants were 12 months old, a statistically significant PLS regression model was still seen, but it was weaker (cross validated predictive power (Q^2^) of 30%), and the set of species associated with having been breast-fed from birth to at least 6 months of age versus formula-fed differed from those seen at 4 months of age (S3 Table).

Being delivered by Caesarean section was modestly associated (PLS correlation <0.1) with being in the SF group at both 4 and 12 months of age. To evaluate if mode of delivery distorted the results the PCA and PLS regressions were repeated for infants that were vaginally born (n = 205). The new models were slightly weaker, but PCA clustering followed the same pattern as seen for all infants and the most influential HOT-taxa associated with being in the EF versus SF group and BFR versus SF group followed the same pattern as when all infants were included (data not shown).

A comparison of the microbiota profile at 4 and 12 months of age for the EF and BFR groups is shown in S1 Fig. The profiles for the SF group (data not shown) were similar to that in the EF group.

## Discussion

The present study compared the oral microbiota of infants fed an experimental formula (EF) supplemented with a bovine MFGM concentrate and a slight reduction in energy and protein content with that of infants fed standard formula (SF) [16]. When comparing the EF and SF groups, the main findings at 4 months of age were that 8 HOT-taxa (3 highly and 5 moderately significant) were characteristic for the EF, while 10 other HOT-taxa were characteristic for the SF group. The species richness did not differ between the two formula-fed groups. Moreover, it was found that the species richness at 4 months was significantly lower in breast-fed than in formula-fed infants.

The most striking finding in the present study was the distinctly different composition of the oral microbiota in the breast-fed compared with the formula-fed infants. This, which is previously well documented for the gut [26, 27] and described for the oral [6] microbiota, may reflect transmission of bacteria from the milk *per se* [28] and/or effects of various milk components on the metabolism or attachment of single bacterial species [27, 29]. Hence, the characteristic presence of bifidobacteria and lactobacilli in the mouth of breast-fed infants that we report is well in line with their presence in milk [30] and the gut of breast-fed infants [31]. It may also be noted that the present metagenomic analysis confirms our previous finding from culturing and colony sequencing of *L*. *gasseri* being one species that characterizes breast-fed versus formula-fed infants [32]. Furthermore, our finding of a lower species richness in breast-fed compared with formula-fed infants is in accordance with results reported for the fecal microbiome [33]. Notably, this difference in species richness was gone when the infants were 12 months old while some differences in the oral microbiota remained even though breast-feeding in most cases was discontinued since several months. This indication of a long-term effect of breast-feeding on the gastrointestinal microbiota deserves to be followed-up.

The MFGM envelops the fat globules in milk and facilitates their secretion from the mammary gland, but it also has important biological functions, including antimicrobial effects *in vitro* and in animal studies [9, 12, 14]. We recently reported that infants fed the MFGM supplemented formula had a lower incidence of AOM and less use of antipyretics [17], and Bruggencate et al [34] found that MFGM intake reduced *Escherichia coli* induced diarrhea in adults. Taken together, these results support a modifying effect of MFGM on the proliferation of certain bacterial species and/or symptoms of infection. The mechanism by which this may occur was not evaluated in the present study, but as the MFGM concentrate is rich in both non-glycosylated and glycosylated bioactive proteins and lipids it may be anticipated that these contribute by modulating the first steps in colonization, i.e. attachment of the bacterial cells and/or their metabolism [6, 35, 36]. In general, there are few studies where the effects of MFGM on infection-related conditions have been studied, and the present study is the first study to show *in vivo* effects on the complex microbiota in the mouth. The results suggest that the findings should be followed-up for potential effects also in the gut.

It may be speculated whether the differences we found in the oral microbiota between the EF and SF groups may relate to the lower incidence of AOM among EF-fed infants up to 6 months of age [17]. The most common bacteria found in the middle ear in otitis are *S*. *pneumoniae*, *H*. *influenzae* and *M*. *catarrhalis* [37]. In this perspective, it is interesting that *M*. *catarrhalis* was less prevalent in oral swabs from the 4 months old EF infants compared to those in the SF group.

One more notable observation in the present study, was that at 12 months of age, the presence of *S*. *mutans*, an opportunistic bacterial species associated with early childhood caries [38], was more prevalent in formula-fed than in breast-fed infants. The association between breast-feeding and risk of caries is debated, but as it is known that earlier colonization by *S*. *mutans* is associated with higher risk for caries development at older age, these results would speak in favor for breast-feeding also in this respect [39]. Previous *in vitro* studies have shown that both human and bovine milk may inhibit the metabolism and adhesion of *S*. *mutans* but results are inconclusive [40, 41]. However, comparisons between formula-fed and breast-fed infants in the present study should be made with caution since these groups were not randomized. There might be unknown group differences correlated to oral microbiota acquisition, i.e. we do not have detailed information on dentation status at 12 months of age.

The strengths of the present study were the double-blind randomized design among formula-fed infants, that sampling was done by trained research nurses and that information on both mode of delivery and use of antibiotics was available. An additional strength was that the obtained sequences could be taxonomically defined against a curated 16S rDNA database specifically built for oral bacteria. The potential sources for bias include selection bias and unbalanced exposure to antibiotics and mode of delivery. Notably, antibiotics had not been given to any of the 4 months-old infants included in the study, and sensitivity analyses in infants that were vaginally delivered confirmed that the overall findings were solid. We cannot exclude that there is a certain degree of selection bias in the study group compared to the general population, but do not think this is significant since allocation to the two formula groups was random and differences in infant growth and microbiota between breast-fed and formula-fed infants are well in line with previous studies [6, 42].

## Conclusions

The microbiota in the oral cavity of infants fed a formula supplemented with MFGM differed from that of infants fed standard formula for a set of taxa. Though the differences between the MFGM-enriched and standard formula were moderate in comparison to the distinct differences between breast-fed and formula-fed infants, the effects may still be of clinical importance. This may be exemplified by the significantly less frequent finding of *M*. *catarrhalis* in the oral cavity and the previously reported lower incidence of acute otitis media in infants fed the EF [17]. However, the impact of MFGM on various infection-associated outcomes needs to be evaluated in larger studies.

## Supporting Information

S1 Checklist CONSORT 2010 checklist. (DOC)

S1 Fig Bilateral bar graph for detection prevalence. Percent infants where a species was found in (A) EF 4 and 12 months old infants, and (B) BFR 4 and 12 months old infants.
(TIF)

S1 Supporting Information Original study protocol. (DOCX)

S1 Table Mean prevalence of phyla and genera sequences. Data are shown in % of all sequences or % of the children where the phylum/genus was detected. Red p-value indicates phyla/genera with a statistically significant difference (p<0,008) between groups.
(PDF)

S2 Table Proportion (% infants) with a species/phylotype detected by sequencing of oral swabs at 4 and 12 months, respectively. EF = experimental formula, SF = standard formula, and BFR = breast-fed reference group. Grey color indicates species/phylotypes in the oral core microbiome.
(PDF)

S3 Table PLS correlation coefficients for taxa with a statistically significant correlation. Statistical significance is indicated by that the 95% CI does not include zero. Text highlighted in grey refers to taxa with a correlation coefficient>0.1 and (G) to the genus level.
(PDF)
