# Supplementary figures and images for: Oral Microbiota in Infants Fed a Formula Supplemented with Bovine Milk Fat Globule Membranes - A Randomized Controlled Trial
Source: PLoS One. 2017 Jan 18;12(1):e0169831. doi: 10.1371/journal.pone.0169831 (PMC5242539; doi:10.1371/journal.pone.0169831)

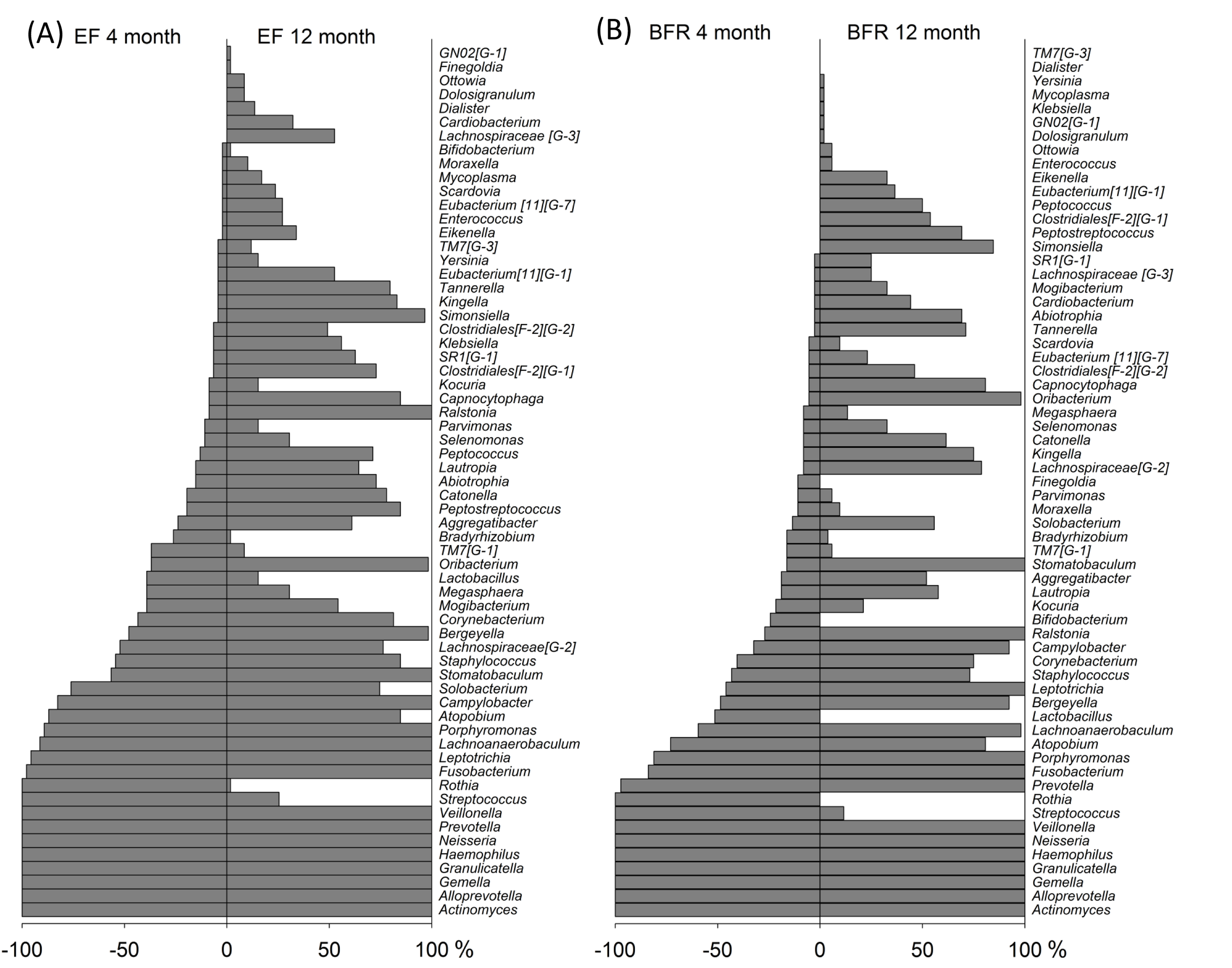

Supplement: S1 Fig — Percent infants where a species was found in (A) EF 4 and 12 months old infants, and (B) BFR 4 and 12 months old infants. (TIF) [file pone.0169831.s002.tif]
