# Supplementary material for: Oral Microbiota in Infants Fed a Formula Supplemented with Bovine Milk Fat Globule Membranes - A Randomized Controlled Trial
Source: PLoS One. 2017 Jan 18;12(1):e0169831. doi: 10.1371/journal.pone.0169831 (PMC5242539; doi:10.1371/journal.pone.0169831)
Supplement: S1 Supporting Information — (DOCX) [file pone.0169831.s003.docx]

Ansökan om etikprövning 2007

Tillväxt och utveckling med ny modersmjölkersättning

**Sammanfattande beskrivning av projektet**

*Bakgrund:*

Bröstmjölk är den bästa födan för spädbarnet under första halvåret. Av olika anledningar kan inte alla barn ammas och man behöver då ge modersmjölkersättning. Trots utvecklingen av modersmjölkersättning finns fortfarande tydliga skillnader mellan ammade och ersättningsuppfödda barn. Man kan visa skillnader i ämnesomsättning och tillväxt som i högre åldrar ger sjuklighet kopplat till dessa ämnesomsättningsfaktorer. Ersättningsuppfödda barn har högre risk att i vuxen ålder drabbas av fetma, nedsatt glukostolerans och ogynnsam blodfettsprofil, alla kända riskfaktorer för hjärtkärlsjukdom Sjuklighet i infektionssjukdomar skiljer sig också mellan ersättningsuppfödda och ammade barn. Ammade barn är mindre sjuka i luftvägsinfektioner, öroninflammationer och magsjuka. Skillnader i psykomotorisk utveckling mellan ammade och ersättningsuppfödda barn har visats i flera stora studier men resultaten har varit omstridda pga de stora svårigheterna att kontrollera socioekonomiska, utbildningsmässiga och ärftliga faktorer som är starkt förknippade med både amning och psykomotorisk utveckling.

Dessa skillnader antas bero på skillnaden i sammansättning av modersmjölkersättning och bröstmjölk. Ersättningsuppfödda barn får i sig ungefär 70% mer protein än ammade barn mellan 3-6 mån ålder vilket ger högre insulinnivåer och ändrat tillväxtmömster. Bröstmjölk innehåller vidare ämnen med antibakteriell, antiviral och immunmodulerande aktivitet som saknas i modersmjölkersättning. En del av dessa är unika för bröstmjölk, medan andra finns i komjölk men försvinner eller förstörs under tillverkningsprocessen. Med ny teknik inom mjölkindustrin har nya fraktioner kunnat renas och finns tillgängliga kommersiellt. En sådan fraktion är ”milk fat globule membrane” (MFGM)

som innehåller ett flertal proteiner med antimikrobiell effekt. MFGM-berikad gröt givet till 6-11 månaders barn har i en nyligen genomförd studie i Peru visats minska incidens och prevalens av diarré.

I vår studie kommer vi att testa en modersmjölksersättning med tillsats av MFGM som har en lägre energitäthet än den konventionella ersättningen.

*Hypootes:*

Barn som får studieersättningen istället för konventionell ersättning kommer att skilja sig mindre från ammade barn när det gäller tillväxt, ämnesomsättningsfaktorer, sjuklighet i infektioner och psykomotorisk utveckling.

*Undersökningsvariabler:*

Neurologisk utveckling: SWEEP-VEP, BSID-II, WPPSI-R

Blod- /avföringsprov: Högkänsligt CRP, IL-6, IL-10, Calprotektin/f, T-RFLP (bakterieflora/f), Calciumtvålar/f, Fettsyraprofil i RBK, Leptin, IGF-1, adiponektin, Insulin + blodsocker 2 tim efter mål, Aminosyrespektrum/b, Urea (BUN)

Antropometri (längd, vikt, hvof, knä-häl)

Blodtryck

Kroppspletysmografi

Kärlförändringar (ultraljud av blodkärl)

*Betydelse:*

Vissa barn kan av olika anledningar inte få bröstmjölk under det första levnadshalvåret. Det är av största vikt att fortsätta förbättringsarbetet med ersättning så att den ökade risk för sjuklighet dessa barn har idag kan minimeras. Varje förbättring kommer att minska sjukligheten i populationen, både under uppväxten men också ha långsiktiga effekter på sjuklighet i vuxen ålder. Teoretiskt finns goda möjligheter att den nya ersättning vi kommer att testa minskar skillnaderna mellan ersättningsuppfödda och ammade barn vad gäller såväl tillväxt, ämnesomsättningsfaktorer, infektionskänslighet och neurologisk utveckling.

**Primär vetenskaplig frågeställning**

Barn som får modersmjölksersättning med tillsats av MFGM samt sänkt energitäthet istället för konventionell modersmjölksersättning kommer att skilja sig mindre från ammade barn när det gäller tillväxt, ämnesomsättningsfaktorer, sjuklighet i infektioner och psykomotorisk utveckling.

**Undersökningsprocedur och datainsamling**

Tre grupper studeras:

1) Barn som får ny ersättning

2) Barn som får konventionell ersättning

3) Barn som ammas helt

*Intervention*

Kostintervention enligt de tre grupperna ovan äger rum från inklusion till 6 månaders ålder. De två ersättningarna blindas och randomiseras så att varken föräldrar, personal eller studieledare känner till vilket barn som får vilken ersättning. Föräldrarna rekommenderas att inte introducera annan kost än ersättning/bröstmjölk innan 6 mån ålder förutom smakportioner (puré av rotsaker och frukt < 1 msk/dag mellan 4-6 mån). Avrådes också från probiotika innan 6 mån ålder.

*Kostregistrering*

Kostdagbok (konsumtion av produkt) förs 3 dygn varje månad från inklusion till 6 månaders ålder för barnen i grupp 1 och 2. Vid inklusion registreras tidigare intag av bröstmjölk och annan föda. Kontinuerligt registreras för alla 3 grupperna intag av annan föda, medicin och AD-droppar.

*Sjukdoms-/symptomregistrering:*

Symptomdagbok förs kontinuerligt från tid för inklusion. Avföringens frekvens och konsistens registreras till 6 månaders ålder, dagar med infektionssymptom (feber >38 grader, snuva, hosta, kräkningar, diarré) registreras till 12 månaders ålder,.

*Provtagning:*

Se punkt 2:1. För varje barn innebär det blodprov som tas via perifer ven i armen vid start,4 ,6 ,12 månaders samt 5 års ålder, Avföringsprov vid start, 4, 6, 12 månaders ålder. SWEEP-VEP vid 6 mån, utvecklingsbedömning vid 12 mån och 5 år, PeaPod vid start och 4 mån samt ultraljud av kärl vid 6 mån och 5 år. Antropometri och blodtryck vid start, 4, 6, 12 mån och 5 år. Besök inklusive blod- och avföringsprov samt antropometri och blodtryck kan beräknas ta 30 min. SWEEP-VEP, Peapod och ultraljudsundersökning av kärl tar ca 30 min vardera och utvecklingsbedömning ca 60 min.

**Urval av forskningspersoner**

*Inklusion*

Barn med en födelsevikt 2500 gram -4500 gram och ålder 0-2 månader vars föräldrar ämnar amma exklusivt till åtminstone 6 mån ålder respektive ge endast ersättning från en tidpunkt max 2 månader efter födseln rekryteras dels via BVC, dels via en telefonenkät till alla föräldrar med 1 mån gamla barn. För att kunna rekrytera tillräckligt många barn inom en rimlig tid (ca 2 år) behöver vi aktivt leta efter barn som kan inkluderas för att fråga föräldrarna om medverkan. I telefonenkäten ställer vi en fråga till föräldrarna om hur barnet föds upp. Om barnet ammas helt eller inte ammas alls, tillfrågas föräldrarna om medverkan i studien. Om barnet ammas delvis ber vi att få återkomma om 2 veckor med samma fråga igen. Största vikt läggs vid att denna telefonfråga ställs neutralt för att inte påverka föräldrarna i uppfödningen av deras barn.

*Exklusionskriterier*

• Kronisk sjukdom som kan påverka utfallsvariablerna, t ex neurologisk, endokrin eller malabsorptionssjukdom

Även barn som av olika anledningar inte har kunnat fullfölja interventionen kommer att följas och inkluderas i den statistiska analysen enligt principen ”intention to treat”.

**Statistiskt underlag för studiepopulationens storlek**

Vi vill kunna påvisa en eventuell skillnad motsvarande 0,5 SD för varje utfallsvariabel, vilket motsvarar en viktskillnad på ca 0,4 kg vid 6 månaders ålder eller en skillnad på 3,25 procentandelar kroppsfett mätt med pletysmografi vid 2 mån ålder. I synskärpa motsvarar 0,5 SD 0,25 oktaver vid 4 mån ålder, vilket är den skillnad man sett när man jämfört barn som fått uppfödning med eller utan DHA. Med en statistisk ”power” på 80% behövs en gruppstorlek av drygt 60 barn. Vi avser att rekrytera 80 barn per grupp, vilket med tidigare bortfallsfrekvens bör ge oss tillräckligt antal barn per grupp som fullföljer studien.

**Information och samtycke**

Skriftlig information delas ut/skickas hem i samband med rekrytering. Muntlig information ges av forskningssjuksköterskan till föräldrarna vid första besöket..

Samtycke till deltagande i studien inhämtas från målsman av forskningssjuksköterska skriftligt vid inklusionsbesöket.

**Forskningsetiska överväganden**

Blodprovstagning av försökspersonerna kan ge lokal smärta vid insticksstället. Innan provtagning bedövas huden med EMLA. Proverna tas av sjuksköterska med stor erfarenhet av blodprovstagning på barn. Alla övriga procedurer är ej invasiva och skall ej ge obehag för barnet. Ingen av undersökningsmetoderna innebär några risker för barnet och den studerade modersmjölksersättningens sammansättning ligger helt inom de aktuella riktlinjerna och skall därmed inte innebära någon risk för att inte ge fullgod näring för barnen i den gruppen.

Om vår hypotes stämmer så kommer de försökspersoner som ingår i gruppen som får den nya ersättningen att få en mer optimal nutrition upp till 6 månaders ålder och därmed en minskad risk för olika tillstånd enl ovan. Den förväntade effekten på det enskilda barnet är dock liten, det är vid jämförelse av större grupper man ev kan se en positiv effekt.

Risken för negativa effekter är försumbar bortsett från obehag vid blodprovstagning. Nyttan för det enskilda barnet är ingen för barnen i kontrollgrupperna som ammas respektive får konventionell ersättning. Barnen som får ny ersättning kan få en mer optimal nutrition

Om den nya ersättningen visar sig vara bättre än den konventionella ersättningen kan det leda till att rekommendationer om modersmjölksersättning kan komma att ändras.

2009-09-16

**Komplettering av tidigare godkänd ansökan dnr 07-083M**

Nitrat och nitrit intas via föda. Nitrat reduceras till nitrit av munhålans bakterier. Nitrit i sin tur omvandlas i kroppen till kväveoxid (NO) som har kärlavslappnande och därmed blodtryckssänkande effekt. Nitrat och nitrit intaget via föda kan därmed teoretiskt ha en hälsofrämjande effekt (Hord NG, Tang Y, Bryan NS: Food sources of nitrates and nitrites: the physiologic context for potential health benefits, Am J Clin Nutr 2009;90:1-10). Mycket lite eller inget av detta finns studerat hos spädbarn. Nitrat- och nitritintag skiljer sig sannolikt mellan ammade och modersmjölkersättningsuppfödda barn beroende på varierande nitrat- och nitritnivåer i dricksvattnet. I vår studie innehåller studieformulan MFGM som kan ha betydelse för munflorans utveckling och därmed ge en skillnad i reduktionen av nitrat till nitrit.

**Vi vill komplettera vår tidigare godkända ansökan med att analysera koncentration av nitrat och nitrit samt bakterieflora med konventionell odling och PCR-metod av bakterie-DNA i saliv hos försökspersonerna vid 4,6 och 12 månaders ålder samt vid 3 och 5 års ålder.** Denna ändring förväntas inte leda till något ökat obehag för försökspersonerna. Information om analyserna har lagts till i föräldrainformationen (bifogas).

Study protocol sent for ethical approval 2007

Growth and development with a new infant formula

**Description of the project**

*Background:*

Human milk is the optimal nutrition during the first year of life. If this is not available, infant formula is necessary. Despite advances in the development of infant formulas, there are still differences between breastfed and formula-fed infants. Metabolic differences and differences in growth may affect morbidity in adult life. Formula-fed infants have higher risk of obesity, impaired glucose tolerance and a non-favorable blood lipid profile, all well-known risk factors for cardiovascular disease. Formula-fed and breastfed infants also differ in risk of infectious diseases. Breastfed infants have a lower risk of airway infections, otitis and gastroenteritis. Several large studies have shown a difference in psychomotor development and cognitive function between breastfed and formula-fed infants. The results are controversial though, because of the problem to control for confounding factors, i.e socioeconomic, educational and hereditary factors. These differences are supposed to be due to differences in the composition between human milk and infant formula. Formula-fed infants have a 70% higher protein intake compared to breastfed infants between 3-6 months of age affecting insulin concentration and growth pattern. Further, human milk contains antibacterial, antiviral and immunomodulating factors that are not present in formula. Some of these are unique to human milk, others are present in cow´s milk but are inactivated during processing to infant formula. With new techniques in the dairy industry, new milk fractions with several proteins with antimicrobial effects are available. In a study in Peru, supplementation with MFGM to complementary food between 6-11 months of age reduced the incidence and prevalence of diarrhea.

In the present study, an infant formula supplemented with MFGM and with reduced energy and protein concentrations will be tested.

*Hypoothesis:*

Infants receiving the new study formula compared to infants receiving standard formula will differ less from breast-fed infants regarding growth, metabolic factors, infectious morbidity and psychomotor development.

*Outcome variables:*

Neurodevelopment: SWEEP-VEP, BSID-II, WPPSI-R

Blood/fecal samples: High-sensitive CRP, IL-6, IL-10, Calprotectin/f, T-RFLP (fecal microbiota), Calcium soaps/f, Lipid profile in RBC, Leptin, IGF-1, adiponectin, Insulin + blood glucose 2 hrs after meal, Amino acids in blood, Urea (BUN)

Anthropometry (length, weight, head circumference, knee-heel)

Blood pressure

Body pletysmography

Vessel changes (ultrasound of blood vessels)

*Why is the study important?*

Breast milk is not available for all infants. It is of greatest importance to continue to improve infant formulas and reduce the over-risk of morbidity for formula-fed infants. Theoretically, the new formula tested in the present study might narrow the gap between breastfed and formula-fed infants regarding growth, metabolic factors, infections and neurodevelopment.

**Hypothesis**

Infants receiving formula supplemented with MFGM and lower energy density compared to standard formula will differ less from breastfed infants regarding growth, metabolic factors, infections and neurodevelopment.

**Study protocol**

Three groups are studied:

1. Infants receiving new formula
2. Infants receiving standard formula
3. Breastfed infants

*Intervention*

Food intervention according to the 3 groups will continue from inclusion until 6 months of age. The study formulas are blinded and randomized, neither parents, staff or investigators are aware of which infant receives which formula. Parents are recommended not to add complementary foods to the infant’s diet before 6 months of age except taste portions (< 1 spoon of vegetables and fruit daily between 4-6 months). Parents are also recommended not to give probiotics before 6 months of age.

*Food diaries*

Parents are asked to record intakes in a 3-day food diary every month from inclusion until 6 months of age for infants in group 1 and 2. At inclusion, previous intakes of breast milk and other foods are recorded. Intakes of food other than breast milk/formula, medication and vitamins are continuously recorded for all infants in the study.

*Disease and symptom diaries*

Parents record any disease symptoms in a symptom diary from inclusion. Fecal frequency and consistency are registered until 6 months of age. Disease symptoms (fever, running nose, coughing, vomiting, diarrhea) are registered until 12 months of age.

*Measurements*

See above. For every infant, a blood test is taken from the arm at inclusion, 4 , 6 and 12 months of age and at 5 years of age. Fecal samples are taken at inclusion, 4 , 6 and 12 months of age. SWEEP-VEP at 6 months. psychological assessment at 12 months and 5 years. PeaPod at inclusion and 4 months. Ultrasound of vessels at 12 months and 5 years. Anthropometry and blood pressure at inclusion, 4, 6, 12 months and 5 years. Visits including blood tests and fecal samples, anthropometry and blood pressure will take approximately 30 minutes. SWEEP-VEP, PeaPod and ultrasound will take approximately 30 minutes each and psychological assessment approximately 60 minutes.

**Study population**

*Inclusion*

Infants with birth weight between 2500-4500 grams and age between 0-2 months and with parents’ intention to exclusively breastfeed or exclusively formula-feed the infant from inclusion until 6 months of age will be redruited from baby health clinics or by telephone contact at 1 month of age. To be able to include the infants in a reasonable time period (2 years), we need to search actively for eligible infants. By telephone, we will ask parents how their infant is fed. If the infant is exclusively breastfed or exclusively formula-fed, parents are asked to join the study. If the infant is partially breastfed, we ask to come back in 2 weeks to ask the same question again. It is of great importance that he telephone question is asked in a neutral way to minimize the risk of affecting the parents’ in the feeding of their infant.

*Exclusion criteria*

Chronic disease that might influence outcome variables, i.e. neurological, endocrine or malabsorption disease.

Children that do not complete the intervention will be followed in the study and included in the analysis according to the principle “intention to treat”.

**Power**

We decide that a difference of 0,5 SD for each main outcome variable is of importance. That corresponds to a 0,4 kg difference in weight at 6 months or a 3,25 %fat difference in body composition at 2 months. For visual acuity, 0,5 SD corresponds to 0,25 octaves at 4 months, which is the difference previously reported between children fed with and without DHA, respectively. In assessment of psychomotor development 0,5 SD corresponds to 7,5 IQ points. With a statistical power of 80% and a level of significance of 0,05, we need a sample size of 60 children in each group. With a drop out frequency similar to our previous studies (approximately 25%), recruitment of 80 children in each group will be sufficient.

**Information and consent**

Written information is given to parents at recruitment. Oral information is given by study nurse at first visit. Written consent is collected from parents at inclusion..

**Ethical considerations**

Blood samples can cause local pain at the injection site. Local anaesthetic (EMLA) is used to minimize this pain. Blood samples are drawn by an experienced nurse. All other procedures are non-invasive and cause no discomfort for the infant. None of the procedures are risky for the infant and the composition of the new study formula is within the present recommendations and regulations for infant formulas and there is no elevated risk for lack of nutrients.

If our hypothesis is correct, the infants allocated to the new formula will have a more optimal nutrition until 6 months of age and a reduced risk of negative health effects as described above. The expected effect in a single infant is small, but may be significant in the comparison between groups.

The risk of negative effects is very small apart from local pain from blood samples. There is no gain for infants in the breast-fed and standard formula groups.

If the new formula is better than standard formula, our results may contribute to new recommendations of composition of infant formulas.

2009-09-16

**Addition to ethical approvement dnr 07-083M**

Nitrate and nitrite is ingested with foods. Nitrate is reduced to nitrite by bacteria in the oral cavity. Nitrite is further metabolized to nitric oxide (NO) with vessel relaxing effects that leads to reduction of the blood pressure. In this way, dietary nitrate and nitrite can yield positive health effects (Hord NG, Tang Y, Bryan NS: Food sources of nitrates and nitrites: the physiologic context for potential health benefits, Am J Clin Nutr 2009;90:1-10). This area is very little studied in infants. Nitrate and nitrite concentrations probably differ between breastfed and formula-fed infants depending on different nitrate and nitrite concentrations in the drinking water. In the present study, the new formula with MFGM might influence the composition of the oral microbiota and affect nitrate to nitrite reduction in the oral cavity.

**We want to add to the previously approved study protocol analysis of nitrate and nitrite concentrations and oral microbiota with culture and PCR-based techniques in saliva from the study persons at 4, 6 and 12 months of age, and at 3 and 5 years of age.** This addition to the study protocol will not cause any discomfort for the infants. Full written and oral information will be given to the parents.
