# Supplementary material for: Oral Microbiota in Infants Fed a Formula Supplemented with Bovine Milk Fat Globule Membranes - A Randomized Controlled Trial
Source: PLoS One. 2017 Jan 18;12(1):e0169831. doi: 10.1371/journal.pone.0169831 (PMC5242539; doi:10.1371/journal.pone.0169831)
Supplement: S1 Table — Data are shown in % of all sequences or % of the children where the phylum/genus was detected. Red p-value indicates phyla/genera with a statistically significant difference (p<0,008) between groups. (PDF) [file pone.0169831.s004.pdf]

S1 Table. Mean prevalence of phyla and genera sequences. Data are shown in % of all sequences or % of the children where the phylum/genus was detected. Red p-value indicates phyla/genera with a statistically significant difference (p<0,008) between groups.

	% of total number of sequenced								% infants with detection							
	4 month				12 month				4 month				12 month			
**Phylum/Genus**	**BFR**	**EF**	**SF**	**p-value**	**BFR**	**EF**	**SF**	**p-value**	**BFR**	**EF**	**SF**	**p-value**	**BFR**	**EF**	**SF**	**p-value**
**Actinobacteria**	**7.850**	**11.230**	**14.660**	**0.057**	**17.898**	**10.270**	**11.824**	**0.001**	100	100	100	1.000	100	100	100	1.000
Actinomyces	1.490	2.720	2.603	0.078	1.611	1.250	1.130	0.088	100	100	100	1.000	100	100	100	1.000
Atopobium	0.268	0.304	0.277	0.972	0.026	0.031	0.027	0.795	73	87	98	0.007	81	85	89	0.484
Bifidobacterium	0.002	0.000	0.000	0.012	0.000	0.000	0.000	0.406	24	2	5	0.001	0	2	0	0.402
Corynebacterium	0.218	0.040	0.036	0.459	0.225	0.244	0.497	0.160	41	43	49	0.757	75	81	86	0.387
Kocuria	0.001	0.003	0.077	0.379	0.018	0.001	0.013	0.494	22	9	20	0.218	21	15	23	0.514
Rothia	5.866	8.164	11.668	0.089	16.018	8.744	10.151	0.001	100	100	100	1.000	100	100	100	1.000
Scardovia	0.002	0.000	0.000	0.18	0.000	0.000	0.000	0.403	5	2	2	0.668	0	2	4	0.368
**Bacteroidetes**	**0.680**	**8.310**	**5.930**	**<0.001**	**3.744**	**6.068**	**6.807**	**0.001**	100	100	100	1.000	100	100	100	1.000
Alloprevotella	0.245	4.907	2.502	<0,001	1.524	2.594	2.179	0.105	100	100	100	1.000	100	100	100	1.000
Bergeyella	0.012	0.009	0.012	0.793	0.057	0.061	0.065	0.804	49	48	51	0.948	92	98	100	0.051
Capnocytophaga	0.000	0.000	0.000	0.957	0.026	0.056	0.057	0.378	5	9	7	0.848	81	85	89	0.484
Porphyromonas	0.152	0.192	0.051	0.166	0.767	1.085	1.219	0.067	81	89	71	0.095	100	100	100	1.000
Prevotella	0.268	3.208	3.366	0.002	1.369	2.271	2.551	0.036	97	100	100	0.306	100	100	100	1.000
Tannerella	0.000	0.000	0.000	0.432	0.000	0.002	0.002	0.241	3	4	0	0.416	12	25	29	0.072
**Firmicutes**	**87.000**	**72.670**	**72.770**	**<0.001**	**58.586**	**59.668**	**61.034**	**0.598**	100	100	100	1.000	100	100	100	1.000
Abiotrophia	0.000	0.000	0.000	0.153	0.127	0.136	0.191	0.442	3	15	7	0.125	69	73	91	0.014
Catonella	0.001	0.002	0.002	0.251	0.006	0.009	0.008	0.291	8	20	27	0.102	62	78	71	0.166
Clostridiales[F-2][G-1]	0.000	0.000	0.001	0.143	0.009	0.015	0.009	0.269	0	7	12	0.091	54	73	51	0.034
Clostridiales[F-2][G-2]	0.000	0.001	0.002	0.125	0.003	0.004	0.005	0.329	5	7	12	0.486	46	49	58	0.427
Dialister	0.000	0.000	0.002	0.018	0.000	0.000	0.000	0.028	0	0	10	0.015	0	14	6	0.015
Dolosigranulum	0.000	0.000	0.000	NA	0.001	0.000	0.000	0.500	0	0	0	1.000	2	9	2	0.128
Enterococcus	0.000	0.000	0.000	0.432	0.000	0.001	0.001	0.082	0	2	0	0.425	6	27	27	0.007
Eubacterium [11][G-7]	0.000	0.000	0.001	0.408	0.004	0.002	0.006	0.374	5	2	15	0.072	23	27	27	0.054
Eubacterium[11][G-1]	0.000	0.002	0.001	0.306	0.007	0.007	0.006	0.953	0	4	12	0.059	37	53	44	0.235
Finegoldia	0.001	0.000	0.001	0.19	0.000	0.000	0.000	0.635	11	0	10	0.079	0	2	2	0.629
Gemella	3.339	2.394	1.956	0.048	3.156	4.445	4.486	0.002	100	100	100	1.000	100	100	100	1.000
Granulicatella	1.214	2.666	1.788	0.028	5.678	5.581	5.598	0.950	100	100	100	1.000	100	100	100	1.000

Lachnoanaerobaculum	0.036	0.192	0.274	0.013	0.218	0.237	0.223	0.883	59	91	98	<0.001	98	100	98	0.572
Lachnospiraceae [G-3]	0.000	0.000	0.000	0.444	0.004	0.005	0.011	0.251	3	0	2	0.547	25	53	36	0.011
Lachnospiraceae[G-2]	0.000	0.077	0.113	0.04	0.021	0.024	0.022	0.888	8	52	56	<0.001	79	76	82	0.769
Lactobacillus	0.012	0.002	0.001	0.092	0.000	0.000	0.000	0.099	51	39	27	0.085	0	15	7	0.011
Megasphaera	0.001	0.016	0.038	0.052	0.001	0.001	0.000	0.129	8	39	51	<0.001	13	31	11	0.014
Mogibacterium	0.000	0.004	0.006	0.009	0.003	0.004	0.004	0.625	3	39	56	<0.001	33	54	44	0.074
Oribacterium	0.001	0.004	0.010	0.048	0.045	0.031	0.045	0.196	5	37	44	<0.001	98	98	89	0.036
Parvimonas	0.000	0.001	0.000	0.268	0.000	0.001	0.002	0.251	11	11	5	0.547	6	15	24	0.036
Peptococcus	0.000	0.007	0.001	0.234	0.016	0.030	0.020	0.144	0	13	7	0.075	50	71	64	0.069
Peptostreptococcus	0.000	0.002	0.003	0.105	0.018	0.017	0.018	0.911	0	20	20	0.016	69	85	78	0.146
Selenomonas	0.000	0.001	0.003	0.289	0.001	0.005	0.002	0.242	8	11	22	0.161	10	24	33	0.050
Solobacterium	0.001	0.040	0.035	0.088	0.043	0.088	0.057	0.071	14	76	90	<0.001	85	97	95	0.046
Staphylococcus	0.007	0.006	0.017	0.533	0.002	0.004	0.004	0.203	43	54	44	0.510	25	63	65	<0.001
Stomatobaculum	0.004	0.009	0.027	0.011	0.035	0.052	0.037	0.295	16	57	61	<0.001	73	85	82	0.285
Streptococcus	79.752	56.984	59.206	<0.001	46.330	45.753	47.209	0.806	100	100	100	1.000	100	100	100	1.000
Veillonella	2.680	10.324	9.279	<0.001	2.861	3.215	3.126	0.692	100	100	100	1.000	100	100	100	1.000
**Fusobacteria**	**1.163**	**1.408**	**1.601**	**0.875**	**2.819**	**4.339**	**3.902**	**0.057**	89	98	98	0.130	100	100	100	1.000
Fusobacterium	0.678	0.430	0.449	0.707	1.630	2.887	2.777	0.033	84	98	95	0.038	100	100	100	1.000
Leptotrichia	0.489	0.977	1.152	0.656	1.189	1.452	1.125	0.564	46	96	95	<0.001	100	100	100	1.000
**GN02**	**0.000**	**0.000**	**0.000**	**NA**	**0.000**	**0.000**	**0.000**	**0.614**	0	0	0	1.000	2	2	5	0.431
GN02[G-1]	0.000	0.000	0.000	NA	0.000	0.000	0.001	0.614	0	0	0	1.000	2	2	5	0.431
**Proteobacteria**	**3.250**	**6.361**	**5.020**	**0.286**	**16.917**	**19.598**	**17.098**	**0.199**	100	100	100	1.000	100	100	100	1.000
Aggregatibacter	0.001	0.003	0.001	0.328	0.031	0.033	0.101	0.035	19	24	22	0.860	52	61	80	0.008
Bradyrhizobium	0.001	0.001	0.001	0.669	0.000	0.000	0.000	0.435	16	26	15	0.341	4	2	6	0.588
Campylobacter	0.014	0.028	0.042	0.181	0.042	0.054	0.050	0.390	32	83	90	<0.001	92	100	98	0.050
Cardiobacterium	0.000	0.000	0.000	0.55	0.016	0.012	0.014	0.759	3	0	2	0.547	44	32	53	0.084
Eikenella	0.000	0.000	0.000	0.579	0.003	0.004	0.004	0.840	0	2	2	0.646	33	34	42	0.559
Haemophilus	1.425	1.251	1.110	0.795	4.199	4.790	5.242	0.136	100	100	100	1.000	100	100	100	1.000
Kingella	0.000	0.000	0.000	0.354	0.067	0.058	0.108	0.243	8	4	0	0.190	75	83	85	0.349
Klebsiella	0.000	0.002	0.000	0.101	0.000	0.004	0.004	<0,001	0	7	0	0.074	2	56	51	<0.001
Lautropia	0.001	0.001	0.000	0.086	0.040	0.068	0.083	0.446	19	15	5	0.153	58	64	84	0.010
Moraxella	0.000	0.000	0.001	0.158	0.004	0.001	0.001	0.490	11	2	12	0.176	10	10	18	0.319
Neisseria	1.810	5.085	3.850	0.197	12.494	14.566	11.468	0.158	100	100	100	1.000	100	100	100	1.000
Ottowia	0.000	0.000	0.000	0.366	0.002	0.002	0.014	0.313	0	0	2	0.360	6	9	15	0.286

Ralstonia	0.001	0.000	0.000	0.045	0.000	0.000	0.000	NA	27	9	10	0.036	0	0	0	1.000
Simonsiella	0.000	0.001	0.009	0.308	0.018	0.006	0.012	0.285	0	4	7	0.258	35	31	33	0.899
Yersinia	0.000	0.001	0.000	0.292	0.000	0.000	0.001	0.076	0	4	0	0.178	2	15	18	0.023
**Spirochaetes**	**0.000**	**0.000**	**0.000**	**NA**	**0.000**	**0.000**	**0.002**	**0.366**	0	0	0	1.000	0	12	7	0.042
Treponema	0.000	0.000	0.000	NA	0.000	0.000	0.002	0.366	0	0	0	1.000	0	12	7	0.042
**SR1**	**0.000**	**0.003**	**0.002**	**0.532**	**0.017**	**0.028**	**0.035**	**0.247**	3	7	10	0.448	56	75	78	0.025
SR1[G-1]	0.000	0.002	0.002	0.532	0.017	0.028	0.035	0.247	3	7	10	0.448	56	75	78	0.025
**Tenericutes**	**0.000**	**0.000**	**0.000**	**0.513**	**0.000**	**0.001**	**0.000**	**0.039**	0	2	2	0.646	2	17	16	0.026
Mycoplasma	0.000	0.000	0.000	0.513	0.000	0.001	0.000	0.039	0	2	2	0.646	2	17	16	0.026
**TM7**	**0.010**	**0.008**	**0.013**	**0.824**	**0.020**	**0.027**	**0.031**	**0.514**	16	39	41	0.033	71	83	76	0.326
TM7[G-1]	0.010	0.008	0.013	0.838	0.020	0.027	0.031	0.521	16	37	39	0.057	71	80	76	0.576
TM7[G-3]	0.000	0.000	0.000	0.132	0.000	0.000	0.000	0.783	0	4	10	0.131	6	9	7	0.860
