# Supplementary material for: Oral Microbiota in Infants Fed a Formula Supplemented with Bovine Milk Fat Globule Membranes - A Randomized Controlled Trial
Source: PLoS One. 2017 Jan 18;12(1):e0169831. doi: 10.1371/journal.pone.0169831 (PMC5242539; doi:10.1371/journal.pone.0169831)
Supplement: S2 Table — EF = experimental formula, SF = standard formula, and BFR = breast-fed reference group. Grey color indicates species/phylotypes in the oral core microbiome. (PDF) [file pone.0169831.s005.pdf]

S2 Table. Proportion (% infants) with a species/phylotype detected by sequencing of oral swabs at 4 and 12 months, respectively. EF=experimental formula, SF=standard formula, and BFR=breast-fed reference group. Grey color indicates species/phylotypes in the oral core microbiome.

Species/phylotype	4 month			12 month			Species/phylotype	4 month			12 month		
(alphabetical order)	BFR	EF	SF	BFR	EF	SF	(sorted by decreasing prevalence)	BFR	EF	SF	BFR	EF	SF
Abiotrophia defectiva	3	15	7	69	73	91	Actinomyces sp. HOT180	97	100	100	98	100	100
Actinomyces graevenitzii	62	93	85	90	97	95	Alloprevotella sp. HOT473	100	100	100	100	98	100
Actinomyces cardiffensis	14	17	20	15	20	25	Gemella haemolysans	100	100	100	100	100	100
Actinomyces johnsonii	3	4	2	31	29	45	Granulicatella elegans	95	100	98	100	100	100
Actinomyces massiliensis	0	0	2	4	2	9	Haemophilus parainfluenzae	97	93	98	100	100	100
Actinomyces meyeri	3	35	37	44	37	29	Neisseria flavescens	95	100	100	100	100	100
Actinomyces naeslundii	3	7	5	42	37	51	Prevotella melaninogenica	97	98	100	100	100	100
Actinomyces naeslundii II	0	0	0	8	8	5	Rothia mucilaginosa	100	100	100	100	100	100
Actinomyces odontolyticus	54	37	44	56	71	69	Streptococcus oligofermentans	100	100	100	100	100	100
Actinomyces oris	11	15	5	60	56	56	Streptococcus oralis	100	100	100	100	100	100
Actinomyces radicidentis	0	0	2	2	0	0	Streptococcus parasanguinis I	97	100	95	100	100	98
Actinomyces sp. HOT170	3	0	0	12	14	15	Streptococcus parasanguinis II	100	100	100	98	97	98
Actinomyces sp. HOT171	0	0	0	19	24	27	Streptococcus peroris	100	100	100	100	100	100
Actinomyces sp. HOT172	49	89	80	98	98	93	Streptococcus pneumoniae	100	100	100	100	100	100
Actinomyces sp. HOT175	0	0	0	21	19	13	Streptococcus salivarius	100	98	100	100	98	100
Actinomyces sp. HOT177	0	9	5	46	25	47	Streptococcus sinensis	100	100	100	96	93	95
Actinomyces sp. HOT178	0	0	0	8	3	5	Streptococcus sp. HOT056	100	100	100	98	95	96
Actinomyces sp. HOT180	97	100	100	98	100	100	Streptococcus sp. HOT058	100	100	100	100	100	100
Actinomyces sp. HOT181	84	100	95	98	97	98	Streptococcus sp. HOT071	100	100	100	100	100	100
Aggregatibacter aphrophilus	0	2	5	12	14	33	Streptococcus sp. HOT074	100	100	100	100	100	100
Aggregatibacter paraphrophilus	0	0	0	4	8	24	Veillonella dispar	100	100	100	96	95	96
Aggregatibacter segnis	14	20	15	21	31	31	Veillonella sp. HOT780	100	100	100	100	100	100
Aggregatibacter sp. HOT458	3	0	2	27	24	44	Veillonella atypica	95	93	98	75	81	71
Aggregatibacter sp. HOT513	5	13	0	8	17	22	Streptococcus australis	92	100	98	100	100	100
Aggregatibacter sp. HOT898	0	0	2	27	31	33	Granulicatella adiacens	92	98	98	100	100	100
Alloprevotella rava	0	0	2	2	17	7	Rothia aeria	92	87	95	100	92	98

Alloprevotella sp. HOT308	24	48	46	52	68	58	Streptococcus sp. HOT064	92	87	85	62	86	82
Alloprevotella sp. HOT473	100	100	100	100	98	100	Streptococcus sp. HOT057	89	98	100	90	86	87
Alloprevotella sp. HOT912	0	11	5	6	19	29	Streptococcus sp. HOT487	86	96	93	100	100	100
Alloprevotella sp. HOT914	16	43	41	94	100	96	Actinomyces sp. HOT181	84	100	95	98	97	98
Alloprevotella tannerae	5	2	10	2	20	15	Gemella sanguinis	84	96	95	98	100	98
Atopobium parvulum	73	87	98	81	85	89	Streptococcus gordonii	81	96	100	96	95	98
Bergeyella sp. HOT322	49	48	51	92	98	100	Porphyromonas sp. HOT279	78	89	71	100	100	100
Bergeyella sp. HOT900	0	4	0	13	32	24	Haemophilus sp. HOT036	78	83	80	96	100	100
Bergeyella sp. HOT907	0	0	7	4	3	15	Fusobacterium periodonticum	73	89	78	100	100	100
Bifidobacterium breve	14	0	2	0	0	0	Atopobium parvulum	73	87	98	81	85	89
Bifidobacterium longum	11	2	2	0	2	0	Rothia dentocariosa	68	63	49	98	98	98
Bradyrhizobium elkanii	16	26	15	4	2	5	Actinomyces graevenitzii	62	93	85	90	97	95
Campylobacter concisus	30	80	90	92	100	95	Neisseria polysaccharea	59	63	68	98	100	95
Campylobacter gracilis	0	4	5	8	8	7	Veillonella parvula	57	91	98	98	95	100
Campylobacter rectus	5	9	10	33	47	51	Prevotella sp. HOT313	57	78	88	54	51	67
Capnocytophaga gingivalis	0	0	2	33	44	47	Fusobacterium nucleatum ss polymorphum	57	70	71	92	97	96
Capnocytophaga granulosa	0	0	0	15	14	18	Actinomyces odontolyticus	54	37	44	56	71	69
Capnocytophaga leadbetteri	3	2	2	44	53	62	Actinomyces sp. HOT172	49	89	80	98	98	93
Capnocytophaga sp. HOT332	0	0	0	0	0	5	Bergeyella sp. HOT322	49	48	51	92	98	100
Capnocytophaga sp. HOT878	0	0	0	6	5	9	Streptococcus sp. HOT066	49	46	59	52	47	51
Capnocytophaga sp. HOT901	0	0	0	4	3	9	Haemophilus haemolyticus	43	46	46	96	90	91
Capnocytophaga sputigena	5	7	2	63	66	65	Streptococcus sp. HOT423	41	83	61	85	80	69
Cardiobacterium hominis	3	0	2	42	31	49	Staphylococcus epidermidis	38	43	41	21	59	60
Cardiobacterium valvulum	0	0	0	8	7	9	Leptotrichia sp. HOT417	35	76	73	94	93	95
Catonella morbi	8	20	27	62	78	71	Lactobacillus gasseri	35	26	22	0	14	7
Clostridiales [F-2][G-1] sp. HOT075	0	7	12	54	73	51	Lactobacillus rhamnosus	35	17	12	0	2	0
Clostridiales [F-2][G-2] sp. HOT085	5	7	12	46	49	58	Lachnoanaerobaculum orale	32	78	83	60	58	58
Corynebacterium diphtheriae	24	33	49	37	39	55	Prevotella sp. HOT299	32	63	56	98	100	100
Corynebacterium durum	11	9	7	62	58	73	Campylobacter concisus	30	80	90	92	100	95
Corynebacterium matruchotii	3	4	0	15	14	16	Lachnoanaerobaculum umeaense	30	78	76	94	100	96
Corynebacterium mucifaciens	8	4	5	2	2	4	Prevotella histicola	30	50	66	27	34	40

Corynebacterium urealyticum	3	2	2	4	2	5
Dialister invisus	0	0	10	0	14	5
Dolosigranulum pigrum	0	0	0	2	8	2
Eikenella corrodens	0	2	2	33	34	42
Enterococcus faecalis	0	2	0	6	27	27
Eubacterium [XI][G-1] sulci	0	4	12	37	53	44
Eubacterium [XI][G-7] yurii	5	2	15	23	27	27
Finegoldia magna	11	0	10	0	2	2
Fusobacterium naviforme	11	0	5	12	22	33
Fusobacterium nucleatum ss polymorphum	57	70	71	92	97	96
Fusobacterium nucleatum ss vincentii	14	20	24	25	36	40
Fusobacterium nucleatum ss. animalis	8	17	24	69	75	76
Fusobacterium nucleatum ss. nucleatum	5	7	7	8	17	31
Fusobacterium periodonticum	73	89	78	100	100	100
Fusobacterium sp. HOT370	19	37	44	85	92	84
Gemella bergeri	0	0	0	6	10	13
Gemella haemolysans	100	100	100	100	100	100
Gemella morbillorum	11	17	12	23	42	53
Gemella sanguinis	84	96	95	98	100	98
GN02 [G-1] sp. HOT872	0	0	0	2	2	5
Granulicatella adiacens	92	98	98	100	100	100
Granulicatella elegans	95	100	98	100	100	100
Haemophilus haemolyticus	43	46	46	96	90	91
Haemophilus influenzae	8	15	15	38	47	60
Haemophilus parainfluenzae	97	93	98	100	100	100
Haemophilus sp. HOT035	22	26	12	88	81	85
Haemophilus sp. HOT036	78	83	80	96	100	100
Haemophilus sp. HOT908	30	39	32	73	83	87
Kingella denitrificans	0	2	0	56	69	73
Kingella kingae	0	0	0	6	5	4
Kingella oralis	8	0	0	33	31	25

Haemophilus sp. HOT908	30	39	32	73	83	87
Neisseria subflava	27	22	22	73	68	75
Ralstonia sp. HOT406	27	9	10	0	0	0
Alloprevotella sp. HOT308	24	48	46	52	68	58
Corynebacterium diphtheriae	24	33	49	37	39	55
Haemophilus sp. HOT035	22	26	12	88	81	85
Kocuria sp. HOT189	22	9	20	21	15	23
Fusobacterium sp. HOT370	19	37	44	85	92	84
Lautropia mirabilis	19	15	5	58	64	84
Veillonella rogosae	16	52	44	85	97	95
Alloprevotella sp. HOT914	16	43	41	94	100	96
TM7 [G-1] sp. HOT352	16	33	34	62	76	64
Bradyrhizobium elkanii	16	26	15	4	2	5
Solobacterium moorei	14	76	90	85	97	95
Prevotella sp. HOT314	14	63	68	62	53	76
Fusobacterium nucleatum ss vincentii	14	20	24	25	36	40
Aggregatibacter segnis	14	20	15	21	31	31
Actinomyces cardiffensis	14	17	20	15	20	25
Bifidobacterium breve	14	0	2	0	0	0
Leptotrichia sp. HOT215	11	67	54	94	100	96
Leptotrichia sp. HOT221	11	54	39	85	83	80
Leptotrichia wadei	11	26	15	21	14	24
Neisseria mucosa	11	17	34	94	95	95
Gemella morbillorum	11	17	12	23	42	53
Staphylococcus caprae	11	17	12	0	3	7
Actinomyces oris	11	15	5	60	56	56
Lachnoanaerobaculum saburreum	11	9	34	10	8	13
Corynebacterium durum	11	9	7	62	58	73
Prevotella oris	11	9	7	2	12	7
Parvimonas micra	11	7	5	4	8	18
Streptococcus intermedius	11	4	2	10	27	35

Kingella sp. HOT012	0	0	0	15	25	29	Bifidobacterium longum	11	2	2	0	2	0
Kingella sp. HOT459	0	4	0	21	37	42	Finegoldia magna	11	0	10	0	2	2
Klebsiella pneumoniae	0	7	0	2	56	51	Fusobacterium naviforme	11	0	5	12	22	33
Kocuria sp. HOT189	22	9	20	21	15	23	Stomatobaculum sp. HOT419	8	52	59	13	14	29
Lachnoanaerobaculum orale	32	78	83	60	58	58	Lachnospiraceae [G-2] sp. HOT096	8	52	56	75	75	82
Lachnoanaerobaculum umeaense	30	78	76	94	100	96	Prevotella salivae	8	48	66	46	73	56
Lachnoanaerobaculum saburreum	11	9	34	10	8	13	Megasphaera micronuciformis	8	39	51	13	31	11
Lachnospiraceae [G-2] sp. HOT088	0	20	22	12	10	20	Catonella morbi	8	20	27	62	78	71
Lachnospiraceae [G-2] sp. HOT096	8	52	56	75	75	82	Leptotrichia sp. HOT463	8	17	27	50	59	47
Lachnospiraceae [G-3] sp. HOT100	3	0	2	25	53	36	Fusobacterium nucleatum ss. animalis	8	17	24	69	75	76
Lactobacillus gasseri	35	26	22	0	14	7	Haemophilus influenzae	8	15	15	38	47	60
Lactobacillus rhamnosus	35	17	12	0	2	0	Stomatobaculum sp. HOT097	8	11	10	65	80	75
Lactococcus lactis	3	11	7	10	24	29	Staphylococcus aureus	8	7	7	4	7	9
Lautropia mirabilis	19	15	5	58	64	84	Corynebacterium mucifaciens	8	4	5	2	2	4
Leptotrichia hofstadii	0	2	0	2	5	5	Kingella oralis	8	0	0	33	31	25
Leptotrichia hongkongensis	0	20	5	46	44	47	Oribacterium sp. HOT108	5	37	44	98	98	89
Leptotrichia shahii	5	24	29	21	17	33	Leptotrichia sp. HOT462	5	28	17	19	31	24
Leptotrichia sp. HOT212	0	4	5	37	39	55	Leptotrichia shahii	5	24	29	21	17	33
Leptotrichia sp. HOT215	11	67	54	94	100	96	Aggregatibacter sp. HOT513	5	13	0	8	17	22
Leptotrichia sp. HOT218	0	9	7	13	8	11	Campylobacter rectus	5	9	10	33	47	51
Leptotrichia sp. HOT219	0	0	0	12	12	11	Clostridiales [F-2][G-2] sp. HOT085	5	7	12	46	49	58
Leptotrichia sp. HOT221	11	54	39	85	83	80	Streptococcus anginosus	5	7	12	0	15	13
Leptotrichia sp. HOT223	0	0	2	0	0	0	Prevotella pallens	5	7	10	19	53	36
Leptotrichia sp. HOT225	0	9	12	38	51	51	Fusobacterium nucleatum ss. nucleatum	5	7	7	8	17	31
Leptotrichia sp. HOT392	0	11	29	73	86	93	Capnocytophaga sputigena	5	7	2	63	66	65
Leptotrichia sp. HOT417	35	76	73	94	93	95	Staphylococcus warneri	5	7	2	0	2	5
Leptotrichia sp. HOT462	5	28	17	19	31	24	Eubacterium [XI][G-7] yurii	5	2	15	23	27	27
Leptotrichia sp. HOT463	8	17	27	50	59	47	Moraxella catarrhalis	5	2	12	10	8	15
Leptotrichia sp. HOT498	0	15	5	0	7	2	Alloprevotella tannerae	5	2	10	2	20	15
Leptotrichia sp. HOT879	0	2	5	31	41	53	Neisseria pharyngis	5	2	7	29	32	33
Leptotrichia wadei	11	26	15	21	14	24	Selenomonas sp. HOT146	5	2	7	2	2	2

Megasphaera micronuciformis	8	39	51	13	31	11	TM7 [G-1] sp. HOT348	5	2	2	0	7	9
Mogibacterium neglectum	3	39	56	33	54	44	Scardovia wiggsiae	5	2	2	0	2	4
Moraxella catarrhalis	5	2	12	10	8	15	Moraxella osloensis	5	0	2	0	2	4
Moraxella osloensis	5	0	2	0	2	4	Mogibacterium neglectum	3	39	56	33	54	44
Mycoplasma salivarium	0	2	2	2	17	16	Actinomyces meyeri	3	35	37	44	37	29
Neisseria elongata	0	2	0	35	56	69	Prevotella veroralis	3	17	17	6	20	9
Neisseria flavescens	95	100	100	100	100	100	Veillonella sp. HOT917	3	15	27	4	2	4
Neisseria lactamica	0	0	10	2	8	13	Abiotrophia defectiva	3	15	7	69	73	91
Neisseria mucosa	11	17	34	94	95	95	Prevotella sp. HOT309	3	13	12	6	27	22
Neisseria oralis	0	0	2	40	51	76	Prevotella oulorum	3	11	17	2	14	18
Neisseria pharyngis	5	2	7	29	32	33	Lactococcus lactis	3	11	7	10	24	29
Neisseria polysaccharea	59	63	68	98	100	95	SR1 [G-1] sp. HOT875	3	7	10	56	75	78
Neisseria sicca	0	0	0	29	36	38	Actinomyces naeslundii	3	7	5	42	37	51
Neisseria sp. HOT015	0	0	0	2	8	7	Prevotella intermedia	3	4	5	4	8	4
Neisseria sp. HOT016	0	2	2	0	8	13	Actinomyces johnsonii	3	4	2	31	29	45
Neisseria sp. HOT018	3	2	7	8	15	9	Corynebacterium matruchotii	3	4	0	15	14	16
Neisseria sp. HOT020	0	0	0	8	10	7	Tannerella sp. HOT286	3	4	0	8	8	22
Neisseria sp. HOT499	0	0	0	2	3	5	Neisseria sp. HOT018	3	2	7	8	15	9
Neisseria subflava	27	22	22	73	68	75	Prevotella denticola	3	2	7	2	7	7
Neisseria weaveri	0	0	0	29	36	36	Capnocytophaga leadbetteri	3	2	2	44	53	62
Oribacterium sp. HOT108	5	37	44	98	98	89	Corynebacterium urealyticum	3	2	2	4	2	5
Ottowia sp. HOT894	0	0	2	6	8	15	Porphyromonas endodontalis	3	2	2	2	3	4
Parvimonas micra	11	7	5	4	8	18	Prevotella shahii	3	0	7	19	34	38
Parvimonas sp. HOT393	0	4	0	2	10	5	Cardiobacterium hominis	3	0	2	42	31	49
Peptococcus sp. HOT168	0	13	7	50	71	64	Aggregatibacter sp. HOT458	3	0	2	27	24	44
Peptostreptococcus stomatis	0	20	20	69	85	78	Lachnospiraceae [G-3] sp. HOT100	3	0	2	25	53	36
Porphyromonas catoniae	0	0	0	8	15	15	Selenomonas noxia	3	0	2	2	2	4
Porphyromonas endodontalis	3	2	2	2	3	4	Actinomyces sp. HOT170	3	0	0	12	14	15
Porphyromonas sp. HOT277	0	2	0	2	5	4	Streptococcus mutans	3	0	0	0	7	11
Porphyromonas sp. HOT278	0	0	0	4	2	9	Lachnospiraceae [G-2] sp. HOT088	0	20	22	12	10	20
Porphyromonas sp. HOT279	78	89	71	100	100	100	Peptostreptococcus stomatis	0	20	20	69	85	78

Porphyromonas sp. HOT284	0	4	0	10	12	16	Leptotrichia hongkongensis	0	20	5	46	44	47
Prevotella denticola	3	2	7	2	7	7	Leptotrichia sp. HOT498	0	15	5	0	7	2
Prevotella histicola	30	50	66	27	34	40	Peptococcus sp. HOT168	0	13	7	50	71	64
Prevotella intermedia	3	4	5	4	8	4	Leptotrichia sp. HOT392	0	11	29	73	86	93
Prevotella melaninogenica	97	98	100	100	100	100	Alloprevotella sp. HOT912	0	11	5	6	19	29
Prevotella oris	11	9	7	2	12	7	Leptotrichia sp. HOT225	0	9	12	38	51	51
Prevotella oulorum	3	11	17	2	14	18	Leptotrichia sp. HOT218	0	9	7	13	8	11
Prevotella pallens	5	7	10	19	53	36	Actinomyces sp. HOT177	0	9	5	46	25	47
Prevotella saccharolytica	0	0	0	2	3	4	Clostridiales [F-2][G-1] sp. HOT075	0	7	12	54	73	51
Prevotella salivae	8	48	66	46	73	56	Prevotella sp. HOT396	0	7	7	17	46	49
Prevotella scopos	0	2	0	15	27	20	Selenomonas sp. HOT136	0	7	2	2	5	5
Prevotella shahii	3	0	7	19	34	38	Klebsiella pneumoniae	0	7	0	2	56	51
Prevotella sp. HOT299	32	63	56	98	100	100	Eubacterium [XI][G-1] sulci	0	4	12	37	53	44
Prevotella sp. HOT305	0	2	7	2	2	2	TM7 [G-3] sp. HOT351	0	4	10	6	8	7
Prevotella sp. HOT309	3	13	12	6	27	22	Selenomonas sp. HOT149	0	4	10	6	7	16
Prevotella sp. HOT310	0	4	2	12	31	29	Simonsiella muelleri	0	4	7	35	31	33
Prevotella sp. HOT313	57	78	88	54	51	67	Leptotrichia sp. HOT212	0	4	5	37	39	55
Prevotella sp. HOT314	14	63	68	62	53	76	Campylobacter gracilis	0	4	5	8	8	7
Prevotella sp. HOT317	0	0	5	2	3	4	Veillonella denticariosi	0	4	5	2	10	5
Prevotella sp. HOT396	0	7	7	17	46	49	Prevotella sp. HOT310	0	4	2	12	31	29
Prevotella veroralis	3	17	17	6	20	9	Kingella sp. HOT459	0	4	0	21	37	42
Ralstonia sp. HOT406	27	9	10	0	0	0	Bergeyella sp. HOT900	0	4	0	13	32	24
Rothia aeria	92	87	95	100	92	98	Porphyromonas sp. HOT284	0	4	0	10	12	16
Rothia dentocariosa	68	63	49	98	98	98	Yersinia pestis	0	4	0	2	15	18
Rothia mucilaginosa	100	100	100	100	100	100	Parvimonas sp. HOT393	0	4	0	2	10	5
Scardovia wiggsiae	5	2	2	0	2	4	Prevotella sp. HOT305	0	2	7	2	2	2
Selenomonas infelix	0	2	0	2	5	0	Leptotrichia sp. HOT879	0	2	5	31	41	53
Selenomonas noxia	3	0	2	2	2	4	Aggregatibacter aphrophilus	0	2	5	12	14	33
Selenomonas sp. HOT126	0	0	0	2	2	2	Selenomonas sp. HOT478	0	2	5	0	5	2
Selenomonas sp. HOT136	0	7	2	2	5	5	Eikenella corrodens	0	2	2	33	34	42
Selenomonas sp. HOT146	5	2	7	2	2	2	Mycoplasma salivarium	0	2	2	2	17	16

Selenomonas sp. HOT149	0	4	10	6	7	16
Selenomonas sp. HOT478	0	2	5	0	5	2
Selenomonas sp. HOT892	0	0	0	0	2	7
Selenomonas sputigena	0	0	2	0	8	7
Simonsiella muelleri	0	4	7	35	31	33
Solobacterium moorei	14	76	90	85	97	95
SR1 [G-1] sp. HOT875	3	7	10	56	75	78
Staphylococcus aureus	8	7	7	4	7	9
Staphylococcus caprae	11	17	12	0	3	7
Staphylococcus epidermidis	38	43	41	21	59	60
Staphylococcus warneri	5	7	2	0	2	5
Stomatobaculum sp. HOT097	8	11	10	65	80	75
Stomatobaculum sp. HOT419	8	52	59	13	14	29
Streptococcus agalactiae	0	0	7	0	2	0
Streptococcus anginosus	5	7	12	0	15	13
Streptococcus australis	92	100	98	100	100	100
Streptococcus gordonii	81	96	100	96	95	98
Streptococcus intermedius	11	4	2	10	27	35
Streptococcus mutans	3	0	0	0	7	11
Streptococcus oligofermentans	100	100	100	100	100	100
Streptococcus oralis	100	100	100	100	100	100
Streptococcus parasanguinis I	97	100	95	100	100	98
Streptococcus parasanguinis II	100	100	100	98	97	98
Streptococcus peroris	100	100	100	100	100	100
Streptococcus pneumoniae	100	100	100	100	100	100
Streptococcus salivarius	100	98	100	100	98	100
Streptococcus sinensis	100	100	100	96	93	95
Streptococcus sp. HOT056	100	100	100	98	95	96
Streptococcus sp. HOT057	89	98	100	90	86	87
Streptococcus sp. HOT058	100	100	100	100	100	100
Streptococcus sp. HOT064	92	87	85	62	86	82

Neisseria sp. HOT016	0	2	2	0	8	13
Kingella denitrificans	0	2	0	56	69	73
Neisseria elongata	0	2	0	35	56	69
Prevotella scopos	0	2	0	15	27	20
TM7 [G-1] sp. HOT347	0	2	0	13	12	22
Enterococcus faecalis	0	2	0	6	27	27
Leptotrichia hofstadii	0	2	0	2	5	5
Porphyromonas sp. HOT277	0	2	0	2	5	4
Selenomonas infelix	0	2	0	2	5	0
Neisseria lactamica	0	0	10	2	8	13
Dialister invisus	0	0	10	0	14	5
Bergeyella sp. HOT907	0	0	7	4	3	15
Streptococcus agalactiae	0	0	7	0	2	0
Prevotella sp. HOT317	0	0	5	2	3	4
Neisseria oralis	0	0	2	40	51	76
Capnocytophaga gingivalis	0	0	2	33	44	47
Aggregatibacter sp. HOT898	0	0	2	27	31	33
Ottowia sp. HOT894	0	0	2	6	8	15
TM7 [G-1] sp. HOT353	0	0	2	6	7	16
Actinomyces massiliensis	0	0	2	4	2	9
Alloprevotella rava	0	0	2	2	17	7
Actinomyces radicidentis	0	0	2	2	0	0
Selenomonas sputigena	0	0	2	0	8	7
Leptotrichia sp. HOT223	0	0	2	0	0	0
Neisseria sicca	0	0	0	29	36	38
Neisseria weaveri	0	0	0	29	36	36
Actinomyces sp. HOT175	0	0	0	21	19	13
Actinomyces sp. HOT171	0	0	0	19	24	27
Kingella sp. HOT012	0	0	0	15	25	29
Capnocytophaga granulosa	0	0	0	15	14	18
Leptotrichia sp. HOT219	0	0	0	12	12	11

Streptococcus sp. HOT066	49	46	59	52	47	51	Porphyromonas catoniae	0	0	0	8	15	15
Streptococcus sp. HOT071	100	100	100	100	100	100	Neisseria sp. HOT020	0	0	0	8	10	7
Streptococcus sp. HOT074	100	100	100	100	100	100	Actinomyces naeslundii II	0	0	0	8	8	5
Streptococcus sp. HOT423	41	83	61	85	80	69	Cardiobacterium valvulum	0	0	0	8	7	9
Streptococcus sp. HOT487	86	96	93	100	100	100	Actinomyces sp. HOT178	0	0	0	8	3	5
Tannerella sp. HOT286	3	4	0	8	8	22	Tannerella sp. HOT808	0	0	0	6	17	11
Tannerella sp. HOT808	0	0	0	6	17	11	Gemella bergeri	0	0	0	6	10	13
TM7 [G-1] sp. HOT346	0	0	0	0	3	4	Capnocytophaga sp. HOT878	0	0	0	6	5	9
TM7 [G-1] sp. HOT347	0	2	0	13	12	22	Kingella kingae	0	0	0	6	5	4
TM7 [G-1] sp. HOT348	5	2	2	0	7	9	Aggregatibacter paraphrophilus	0	0	0	4	8	24
TM7 [G-1] sp. HOT352	16	33	34	62	76	64	Capnocytophaga sp. HOT901	0	0	0	4	3	9
TM7 [G-1] sp. HOT353	0	0	2	6	7	16	Porphyromonas sp. HOT278	0	0	0	4	2	9
TM7 [G-3] sp. HOT351	0	4	10	6	8	7	Neisseria sp. HOT015	0	0	0	2	8	7
Treponema vincentii	0	0	0	0	12	7	Dolosigranulum pigrum	0	0	0	2	8	2
Veillonella atypica	95	93	98	75	81	71	Neisseria sp. HOT499	0	0	0	2	3	5
Veillonella denticariosi	0	4	5	2	10	5	Prevotella saccharolytica	0	0	0	2	3	4
Veillonella dispar	100	100	100	96	95	96	GN02 [G-1] sp. HOT872	0	0	0	2	2	5
Veillonella parvula	57	91	98	98	95	100	Selenomonas sp. HOT126	0	0	0	2	2	2
Veillonella rogosae	16	52	44	85	97	95	Treponema vincentii	0	0	0	0	12	7
Veillonella sp. HOT780	100	100	100	100	100	100	TM7 [G-1] sp. HOT346	0	0	0	0	3	4
Veillonella sp. HOT917	3	15	27	4	2	4	Selenomonas sp. HOT892	0	0	0	0	2	7
Yersinia pestis	0	4	0	2	15	18	Capnocytophaga sp. HOT332	0	0	0	0	0	5
