# Supplementary material for: Oral Microbiota in Infants Fed a Formula Supplemented with Bovine Milk Fat Globule Membranes - A Randomized Controlled Trial
Source: PLoS One. 2017 Jan 18;12(1):e0169831. doi: 10.1371/journal.pone.0169831 (PMC5242539; doi:10.1371/journal.pone.0169831)
Supplement: S3 Table — Statistical significance is indicated by that the 95% CI does not include zero. Text highlighted in grey refers to taxa with a correlation coefficient>0.1 and (G) to the genus level. (PDF) [file pone.0169831.s006.pdf]

S3 Table. PLS correlation coefficients for taxa and biological covariates with a statistically significant correlation. Statistical significance is indicated by that the PLS correlation coeff (Corr) 95% CI does not include zero. Text highlighted in grey refers to taxa with a correlation coefficient >0.1 and (G) to the genus level. Q^2=cross validated predicitve value of the model.

EF versus SF group						BFR versus no BFR					
4 month (Q2=0.15)			12 month (Q2=0.05)			4 month (Q2=0.57)			12 month (Q2=0.30)		
**EF**	**Factor**	Corr	**EF**	**Factor**	Corr	**BFR**	**Factor**	Corr	**BFR**	**Factor**	Corr
	Aggregatibacter sp. HOT513	0.140		Megasphaera (G)	0.138	**yes**	Lactobacillus (culture)	0.116	**yes**	Rothia aeria	0.063
	Streptococcus sp. HOT423	0.132		Megasphaera micronuciformis	0.138		Bifidobacterium (G)	0.106		Streptococcus sp. HOT423	0.055
	Porphyromonas (G)	0.126		Clostridiales [F-2][G-1] sp. HOT075	0.124		Bifidobacterium breve	0.086		Oribacterium (G)	0.044
	Porphyromonas sp. HOT279	0.126		Clostridiales[F-2][G-1] (G)	0.124		Kingella oralis	0.079		Oribacterium sp. HOT108	0.044
	Klebsiella (G)	0.097		Oribacterium (G)	0.107		Ralstonia (G)	0.076		Actinomyces sp. HOT172	0.030
	Klebsiella pneumoniae	0.097		Oribacterium sp. HOT108	0.107		Ralstonia sp. HOT406	0.076			
	Actinomyces oris	0.093		Prevotella pallens	0.096		Lactobacillus rhamnosus	0.074			
	Rothia dentocariosa	0.078		Neisseria polysaccharea	0.095		Lactobacillus (G)	0.055			
	Leptotrichia wadei	0.077		Prevotella salivae	0.094		Sex	0.040			
	Abiotrophia (G)	0.068		Lachnospiraceae [G-3] (G)	0.087		Lactobacillus gasseri	0.037			
	Abiotrophia defectiva	0.068		Lachnospiraceae [G-3] sp. HOT100	0.087						
	Sex	0.021		Dolosigranulum (G)	0.085	**no**			**no**		
				Dolosigranulum pigrum	0.085		Solobacterium (G)	-0.214		Klebsiella (G)	-0.235
				Alloprevotella rava	0.085		Solobacterium moorei	-0.214		Klebsiella pneumoniae	-0.235
				Lactobacillus (G)	0.072		Leptotrichia (G)	-0.187		Staphylococcus (G)	-0.172
				Lactobacillus gasseri	0.059		Campylobacter concisus	-0.178		Staphylococcus epidermidis	-0.170
							Campylobacter (G)	-0.176		Prevotella sp. HOT396	-0.139
**SF**			**SF**				Prevotella sp. HOT314	-0.156		Neisseria elongata	-0.122
	Lachnoanaerobaculum saburreum	-0.171		Neisseria oralis	-0.153		Lachnoanaerobaculum orale	-0.152		Prevotella pallens	-0.119
	Dialister (G)	-0.127		Prevotella sp. HOT314	-0.144		Leptotrichia sp. HOT215	-0.151		Streptococcus sp. HOT064	-0.119
	Dialister invisus	-0.127		Aggregatibacter aphrophilus	-0.140		Veillonella parvula	-0.150		Enterococcus (G)	-0.115
	Finegoldia (G)	-0.127		Actinomyces sp. HOT177	-0.136		Lachnoanaerobaculum umeaense	-0.147		Enterococcus faecalis	-0.115
	Finegoldia magna	-0.127		Abiotrophia (G)	-0.133		Prevotella salivae	-0.146		Gemella morbillorum	-0.108
	Neisseria lactamica	-0.127		Abiotrophia defectiva	-0.133		Moryella sp. HOT419	-0.143		Prevotella sp. HOT309	-0.108
	Leptotrichia sp. HOT392	-0.126		Bergeyella sp. HOT907	-0.133		Lachnoanaerobaculum (G)	-0.142		Streptococcus intermedius	-0.107

Eubacterium [11][G-7] (G)	-0.125
Eubacterium [XI][G-7] yurii	-0.125
Streptococcus agalactiae	-0.109
Moraxella (G)	-0.108
Moraxella catarrhalis	-0.108
Atopobium (G)	-0.106
Atopobium parvulum	-0.106
Solobacterium (G)	-0.102
Solobacterium moorei	-0.102
Prevotella salivae	-0.099
Prevotella histicola	-0.087
Selenomonas (G)	-0.082
Campylobacter concisus	-0.075
Lachnoanaerobaculum (G)	-0.073
Veillonella parvula	-0.073
Megasphaera (G)	-0.066
Megasphaera micronuciformis	-0.066

Aggregatibacter paraphrophilus	-0.131
Aggregatibacter sp. HOT458	-0.127
Lautropia (G)	-0.126
Lautropia mirabilis	-0.126
Porphyromonas sp. HOT278	-0.124
Cardiobacterium (G)	-0.123
Aggregatibacter (G)	-0.120
Tannerella sp. HOT286	-0.120
Moryella sp. HOT419	-0.119
Cardiobacterium hominis	-0.114
Selenomonas sp. HOT892	-0.110
Actinomyces johnsonii	-0.104
TM7 [G-1] sp. HOT353	-0.102
Actinomyces massiliensis	-0.092
Fusobacterium nucleatum ss. nucleatum	-0.090
Leptotrichia wadei	-0.085
Leptotrichia sp. HOT212	-0.084
Actinomyces naeslundii	-0.083
Moraxella (G)	-0.063
Clostridiales [F-2][G-2] sp. HOT085	-0.056
Clostridiales[F-2][G-2] (G)	-0.056
Eikenella (G)	-0.053
Eikenella corrodens	-0.053
Prevotella histicola	-0.042

Mogibacterium (G)	-0.141
Mogibacterium neglectum	-0.141
Lachnospiraceae [G-2] sp. HOT096	-0.140
Lachnospiraceae[G-2] (G)	-0.140
Stomatobaculum (G)	-0.127
Actinomyces sp. HOT172	-0.125
Leptotrichia sp. HOT417	-0.123
Megasphaera (G)	-0.116
Megasphaera micronuciformis	-0.116
Oribacterium (G)	-0.114
Oribacterium sp. HOT108	-0.114
Leptotrichia sp. HOT221	-0.113
Actinomyces meyeri	-0.113
Actinomyces graevenitzii	-0.106
Streptococcus sp. HOT423	-0.099
Veillonella rogosae	-0.099
Streptococcus gordonii	-0.096
Prevotella sp. HOT313	-0.090
Lachnospiraceae [G-2] sp. HOT088	-0.088
Leptotrichia sp. HOT392	-0.085
Peptostreptococcus (G)	-0.085
Peptostreptococcus stomatis	-0.085
Actinomyces sp. HOT181	-0.085
Prevotella histicola	-0.083
Alloprevotella sp. HOT914	-0.083
Atopobium (G)	-0.083
Atopobium parvulum	-0.083
Prevotella sp. HOT299	-0.082
Leptotrichia shahii	-0.079
Veillonella sp. HOT917	-0.075

Alloprevotella tannerae	-0.105
Streptococcus anginosus	-0.103
Yersinia (G)	-0.101
Yersinia pestis	-0.101
Alloprevotella sp. HOT912	-0.101
Neisseria oralis	-0.100
Leptotrichia sp. HOT392	-0.099
SR1 [G-1] sp. HOT875	-0.098
SR1[G-1] (G)	-0.098
Mycoplasma (G)	-0.097
Mycoplasma salivarium	-0.097
Prevotella sp. HOT310	-0.096
Selenomonas (G)	-0.096
Lactobacillus (G)	-0.095
Prevotella oulorum	-0.093
Fusobacterium nucleatum ss. nucleatum	-0.092
Lactococcus lactis	-0.092
Lactobacillus gasseri	-0.091
Caesarean	-0.089
Bergeyella (G)	-0.088
Bergeyella sp. HOT322	-0.088
Campylobacter	-0.088
Lachnospiraceae [G-3] (G)	-0.088
Lachnospiraceae [G-3] sp. HOT100	-0.088
Kingella sp. HOT459	-0.088
Dialister (G)	-0.087
Dialister invisus	-0.087
Neisseria sp. HOT016	-0.087
Treponema (G)	-0.087
Treponema vincentii	-0.087

Streptococcus sp. HOT057	-0.074	Parvimonas (G)	-0.085
TM7[G-1] (G)	-0.070	Aggregatibacter	-0.083
Alloprevotella sp. HOT308	-0.070	Prevotella salivae	-0.083
Leptotrichia sp. HOT462	-0.069	Streptococcus mutans	-0.082
Fusobacterium sp. HOT370	-0.067	Prevotella shahii	-0.082
Leptotrichia hongkongensis	-0.067	Alloprevotella rava	-0.081
Prevotella veroralis	-0.065	Fusobacterium naviforme	-0.081
Leptotrichia sp. HOT225	-0.060	Aggregatibacter paraphrophilus	-0.078
Leptotrichia sp. HOT498	-0.060	Mogibacterium (G)	-0.075
Peptococcus (G)	-0.060	Mogibacterium neglectum	-0.075
Peptococcus sp. HOT168	-0.060	Lautropia (G)	-0.075
Streptococcus australis	-0.059	Lautropia mirabilis	-0.075
Caesarean	-0.058	Bergeyella sp. HOT900	-0.074
Catonella (G)	-0.057	Selenomonas sputigena	-0.073
Catonella morbi	-0.057	TM7 [G-1] sp. HOT348	-0.073
TM7 [G-1] sp. HOT352	-0.057	Aggregatibacter sp. HOT513	-0.072
Clostridiales [F-2][G-1] sp. HOT075	-0.056	Neisseria lactamica	-0.072
Clostridiales[F-2][G-1] (G)	-0.056	Campylobacter rectus	-0.072
Prevotella oulorum	-0.054	Actinomyces odontolyticus	-0.070
Leptotrichia sp. HOT463	-0.054	Kingella denitrificans	-0.070
Neisseria mucosa	-0.053	Parvimonas micra	-0.069
Alloprevotella sp. HOT912	-0.052	Haemophilus influenzae	-0.065
Prevotella sp. HOT309	-0.050	Staphylococcus caprae	-0.063
Corynebacterium diphtheriae	-0.050	Prevotella oris	-0.062
Actinomyces sp. HOT177	-0.048	Fusobacterium nucleatum ss vincentii	-0.061
Prevotella sp. HOT396	-0.048	Aggregatibacter aphrophilus	-0.060
Selenomonas sp. HOT149	-0.048	Prevotella veroralis	-0.058
TM7 [G-3] sp. HOT351	-0.048	Tannerella sp. HOT808	-0.058
TM7[G-3] (G)	-0.048	Veillonella denticariosi	-0.056
Simonsiella (G)	-0.044	Capnocytophaga leadbetteri	-0.055
Simonsiella muelleri	-0.044	Leptotrichia sp. HOT498	-0.051

Fusobacterium periodonticum	-0.041	Selenomonas sp. HOT478	-0.051
Campylobacter gracilis	-0.039	Selenomonas sp. HOT892	-0.051
Dialister (G)	-0.039	Staphylococcus warneri	-0.051
Dialister invisus	-0.039	TM7 [G-1] sp. HOT346	-0.051
Neisseria lactamica	-0.039	Neisseria sp. HOT015	-0.050
Prevotella sp. HOT305	-0.039	Prevotella denticola	-0.050
Selenomonas sp. HOT136	-0.039	Corynebacterium diphtheriae	-0.046
Lachnoanaerobaculum saburreum	-0.039	Prevotella scopos	-0.046
Klebsiella (G)	-0.034	Moraxella osloensis	-0.044
Klebsiella pneumoniae	-0.034	Scardovia (G)	-0.044
Leptotrichia sp. HOT879	-0.034	Scardovia wiggsiae	-0.044
Prevotella sp. HOT310	-0.034		
Selenomonas sp. HOT478	-0.034		
Streptococcus agalactiae	-0.034		
SR1 [G-1] sp. HOT875	-0.033		
SR1[G-1] (G)	-0.033		
Fusobacterium nucleatum ss vincentii	-0.032		
